# VEGFR2 pY949 signalling regulates adherens junction integrity and metastatic spread

**DOI:** 10.1038/ncomms11017

**Published:** 2016-03-23

**Authors:** Xiujuan Li, Narendra Padhan, Elisabet O. Sjöström, Francis P. Roche, Chiara Testini, Naoki Honkura, Miguel Sáinz-Jaspeado, Emma Gordon, Katie Bentley, Andrew Philippides, Vladimir Tolmachev, Elisabetta Dejana, Radu V. Stan, Dietmar Vestweber, Kurt Ballmer-Hofer, Christer Betsholtz, Kristian Pietras, Leif Jansson, Lena Claesson-Welsh

**Affiliations:** 1 Department of Immunology, Genetics and Pathology, Rudbeck Laboratory, Science for Life Laboratory, Uppsala University, 751 85 Uppsala, Sweden; 2 Beth Israel Deaconess Medical Center, Harvard Medical School, 330 Brookline Avenue, Boston, Massachusetts 02215, USA; 3 Centre for Computational Neuroscience and Robotics, University of Sussex, Chichester 1 CI 104, Brighton BN1 9RH, UK; 4 c/o IFOM-IEO Campus, Via Adamello, 16, 20139 Milan, Italy; 5 Department of Pathology, Dartmouth College, Geisel School of Medicine at Dartmouth, Lebanon, New Hampshire 03756, USA; 6 Department of Vascular Cell Biology, Max Planck Institute for Molecular Biomedicine, Röntgenstraße 20, 48149 Münster, Germany; 7 Biomolecular Research, Molecular Cell Biology, Paul-Scherrer Institute, 5232 Villigen-PSI, Switzerland; 8 Karolinska Institutet, Dept. Medical Biochemistry and Biophysics, Div. Vascular Biology, 17177 Stockholm, Sweden; 9 Translational Cancer Research, Medicon Village, Lund University, Building 404:A3, 22381 Lund, Sweden; 10 Department of Medical Cell Biology, Biomedical Center, Uppsala University, Box 571, 751 23 Uppsala, Sweden

## Abstract

The specific role of VEGFA-induced permeability and vascular leakage in physiology and pathology has remained unclear. Here we show that VEGFA-induced vascular leakage depends on signalling initiated via the VEGFR2 phosphosite Y949, regulating dynamic c-Src and VE-cadherin phosphorylation. Abolished Y949 signalling in the mouse mutant *Vegfr2*^*Y949F/Y949F*^ leads to VEGFA-resistant endothelial adherens junctions and a block in molecular extravasation. Vessels in *Vegfr2*^*Y949F/Y949F*^ mice remain sensitive to inflammatory cytokines, and vascular morphology, blood pressure and flow parameters are normal. Tumour-bearing *Vegfr2*^*Y949F/Y949F*^ mice display reduced vascular leakage and oedema, improved response to chemotherapy and, importantly, reduced metastatic spread. The inflammatory infiltration in the tumour micro-environment is unaffected. Blocking VEGFA-induced disassembly of endothelial junctions, thereby suppressing tumour oedema and metastatic spread, may be preferable to full vascular suppression in the treatment of certain cancer forms.

## Abstract (web summary)

Signals through VEGF receptor 2 (VEGFR2) increase vascular permeability, promoting cancer progression. Here the authors show that a point mutation in VEGFR2 preventing its auto-phosphorylation leads to reduced metastatic spread and improved response to chemotherapy in tumor-bearing mice, without affecting tumor inflammation.

## Graphical abstract

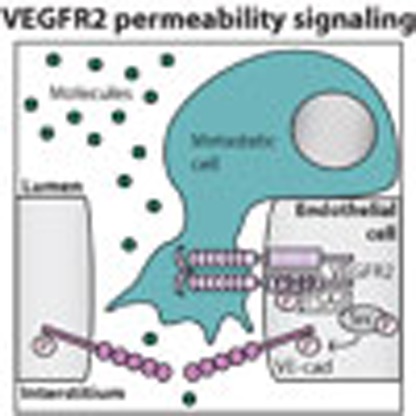

Cancer is characterized by a dysfunctional and morphologically abnormal vasculature with elevated permeability and sparse pericyte and basement membrane coating, resulting in unstable vessels and poor blood flow^1^. The dismal condition of the tumour vasculature is attributed to the hypoxic tumour micro-environment and the accompanying inflammation resulting in the production of a broad range of growth modulatory factors including vascular endothelial growth factors (VEGFs), in particular VEGFA (ref. 2). VEGFA causes increased vascular leakage by disruption of adherens junctions created through homophilic interactions between vascular endothelial (VE)-cadherin molecules expressed on adjacent endothelial cells^3^. Vessel normalization by titrating an appropriate level of anti-VEGFA therapy improves the efficacy of irradiation and chemotherapy^4^. However, sustained anti-VEGFA therapy deteriorates vessel function and may cause increased hypoxia, increased local tumour cell invasion and increased metastatic propensity (for a review, see ref. 5). The role of VEGFA-induced permeability compared with VEGFA-regulated migration, survival and proliferation of endothelial cells is unknown. Moreover, the signal transduction pathways induced by VEGFA to promote increased vascular permeability have remained unidentified.

VEGFA exerts its effects via two endothelial cell receptor tyrosine kinases denoted VEGFR1 and VEGFR2 (ref. 6). VEGFR1 binds and neutralizes VEGFA, thereby exerting a negative regulatory effect on endothelial cells, while VEGFR2 is essential in all known VEGFA biology^6^. VEGFR2 becomes activated and phosphorylated on tyrosine residues in response to VEGFA: Y949, Y1052, Y1057, Y1173 and Y1212 (mouse sequence numbers)^7^. The Y949 phosphosite in VEGFR2 (Y951 in human VEGFR2) presents a specific binding site for the T cell-specific adaptor (TSAd). TSAd is implicated in VEGFA-induced permeability, by regulating VEGFR2-dependent c-Src signalling at endothelial cell junctions^8^. The Y1052/1057 residues, located on the tyrosine kinase activation loop, are required for full kinase activity (see ref. 6 and references therein). The phosphorylated Y1173 binds phospholipase Cγ, of importance for endothelial ERK1/2 pathway activation^9^. A phenylalanine knock-in mouse *Vegfr2*^*Y1173F/Y1173F*^ is embryonically lethal due to arrested endothelial cell development^10^. The *Vegfr2*^*Y1212F/Y1212F*^ mouse lacks a developmental phenotype on a mixed background^10^.

The aim of the current study was to examine the consequence of specific suppression of VEGFA-induced permeability on cancer progression. VEGFA-induced molecular extravasation was attenuated in the *Vegfr2*^*Y949F/Y949F*^ mouse due to perturbed signalling through the TSAd/c-Src/VE-cadherin pathway resulting in VEGFA-resistant adherens junctions. The Y949F mutation however did not perturb other aspects of VEGFA-regulated vessel biology. The loss of VEGFA-regulated leakage was compatible with vascular development and a morphologically normal vasculature including the presence of fenestrated endothelium in adult organs. Blood flow and blood pressure changes in response to VEGFA were unaffected by the mutation. In cancer, the loss of VEGFA-regulated leakage was manifested as reduced tumour oedema, improved responsiveness to chemotherapy and suppressed metastatic spread due to an arrest in tumour cell intravasation.

## Results

### *Vegfr2* ^*Y949F/Y949F*^ vessels do not leak in response to VEGFA

**Figure 1:**
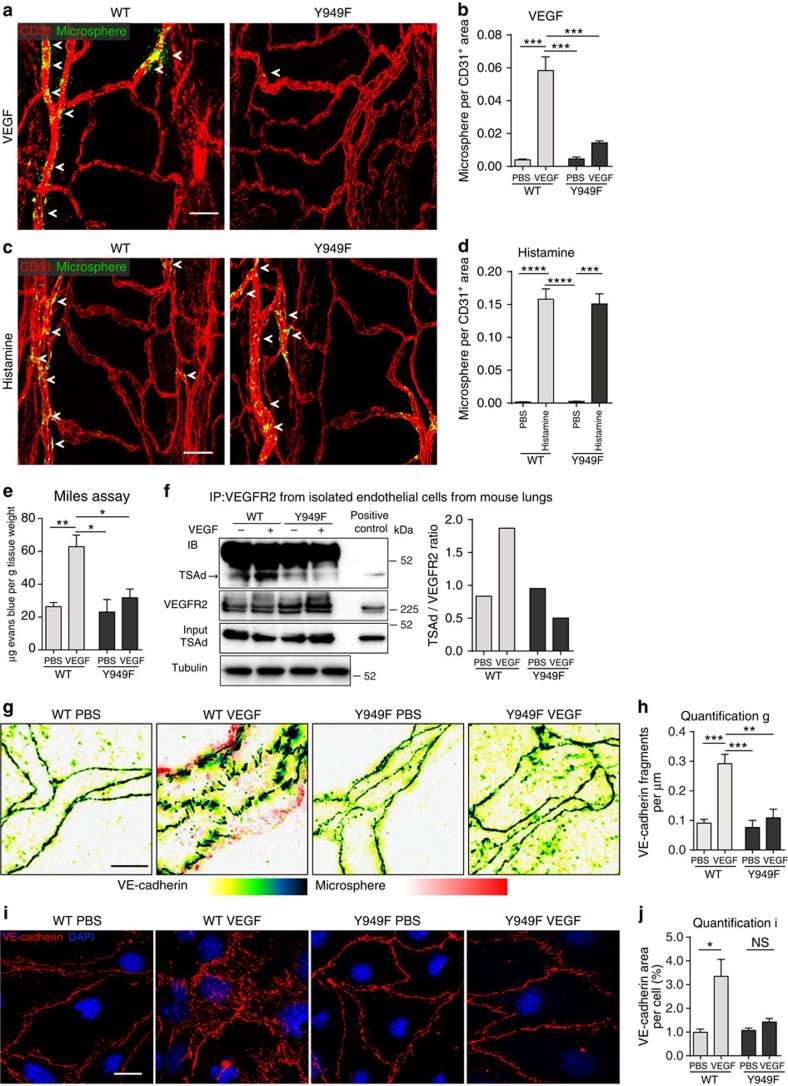
Arrest in VEGFA-induced vascular leakage in *Vegfr2*^*Y949F/Y949F*^ mice. (**a**) Tracheal vessel leakage in WT (left) and *Vegfr2*^*Y949F/Y949F*^ (Y949F; right) mice after tail vein injection of VEGFA or PBS combined with 30 nm fluorescent microspheres (green). Arrows: microsphere leakage from CD31^+^ vessels (red). Scale bar, 50 μm. (**b**) Microsphere area per CD31-positive area fraction in VEGFA/PBS samples from **a**. *n*=3 mice per genotype. Two-way ANOVA: *P*(treatment)<0.0001; *P*(genotype)=0.001; *P*(interaction)=0.0008. (**c**) Tracheal vessel leakage in response to tail vein-injected histamine (as in **a**), in WT and *Vegfr2*^*Y949F/Y949F*^ (Y949F) mice. (**d**) Microsphere area per CD31-positive area fraction in histamine/PBS samples from **c**. *n*=3–6 mice per genotype. Two-way ANOVA: *P*(treatment)<0.0001; *P*(genotype)=0.84; *P*(interaction)=0.80. (**e**) Evans' blue extravasation induced by VEGFA/PBS in the dermis of WT and *Vegfr2*^*Y949F/Y949F*^ (Y949F) mice. Values were normalized to tissue weight. *n*=3 mice per genotype. Two-way ANOVA: *P*(treatment)=0.0053; *P*(genotype)=0.0206; *P*(interaction)=0.0482. (**f**) VEGFR2/TSAd complex formation in isolated mouse lung endothelial cells from WT and *Vegfr2*^*Y949F/Y949F*^ (Y949F) mice, treated or not with VEGFA. Immunoprecipitation (IP) for VEGFR2 and immunoblotting for TSAd and VEGFR2. Total lysates were immunoblotted for TSAd and tubulin. Positive control, total lysate. Molecular weight markers to the right. (**g**) VE-cadherin immunostaining (yellow–black heatmap) on trachea from WT and *Vegfr2*^*Y949F/Y949F*^ (Y949F) mice, tail vein-injected with VEGFA/PBS and microspheres (pink–red heatmap). Images were rendered using structured illumination microscopy. Note VE-cadherin rearrangement in VEGFA-injected WT trachea. Scale bar, 10 μm. (**h**) VE-cadherin fragments per μm vessel length from **g**. *n*=3–6 individual tissues per genotype. Total vessel length assessed: WT/PBS, 236 μm; WT/VEGFA, 308 μm; Y949F/PBS, 150 μm; and Y949F/VEGFA, 145 μm. Two-way ANOVA: *P*(treatment)=0.0022; *P*(genotype)=0.0065; *P*(interaction)=0.0164. (**i**) VE-cadherin immunostaining (red) on endothelial cells isolated from WT and *Vegfr2*^*Y949F/Y949F*^ (Y949F) mouse lungs and then treated or not with VEGFA (100 ng ml^−1^). Hoechst 33342 (blue) staining shows nuclei. Scale bar, 20 μm. (**j**) VE-cadherin-positive staining per cell; samples generated as in *i*. *n*=30 cells per condition. Two-way ANOVA: *P*(treatment)=0.0189; *P*(genotype)=0.099; *P*(interaction)=0.0741. Data presented as mean±s.e.m. Two-way ANOVA with Tukey's *post hoc* test, *P<0.05, **P<0.01, ***P<0.001, ****P<0.0001. Experiments were performed at least three independent times.

*Vegfr2*^*Y949F/Y949F*^ knock-in mice were created using Velocigene technology^11^ and used in this study after selection cassette removal, sequence verification and extensive backcrossing onto the C57Bl/6 background (Supplementary Fig. 1). The mutant mice were phenotypically normal and expressed similar levels of VEGFR2 protein in the vasculature as wild-type (WT) mice (see below). To determine the consequence of *Vegfr2*^*Y949F/Y949F*^ mutation on VEGFA-induced vascular leakage *in vivo*, we employed two well-established assays, microsphere extravasation in the tracheal vasculature and Miles extravasation assay in the dermis. To induce microsphere extravasation, VEGFA or vehicle was administered by tail vein injection together with fluorescent 30-nm microspheres. VEGFA-induced extravasation of microspheres, lodged beneath the basement membrane in postcapillary venules, was significantly reduced at 60 min of circulation in the *Vegfr2*^*Y949F/Y949F*^ trachea compared with WT (Fig. 1a,b). In contrast, tail vein-administered histamine induced similar levels of microsphere extravasation in WT and *Vegfr2*^*Y949F/Y949F*^ trachea venules (Fig. 1c,d). Moreover, VEGFA induced a significant increase of Evans' blue leakage in the skin (Miles assay) in the WT but not in the *Vegfr2*^*Y949F/Y949F*^ mouse (Fig. 1e).

We have previously shown that the phosphorylated Y949 site in VEGFR2 serves as a binding site for Src homology 2 (SH2) domain of TSAd^8^, which in turn binds the SH3 domain of c-Src. c-Src is known to regulate VE-cadherin and adherens junctions integrity^12^. We verified VEGFA-induced VEGFR2/TSAd complex formation in the WT and lack of TSAd binding to mutant VEGFR2-Y949F, by immunoblotting on isolated endothelial cells from WT and *Vegfr2*^*Y949F/Y949F*^ mouse lungs (Fig. 1f).

We next analysed the consequence of the VEGFR2-Y949F mutation of VE-cadherin stability. Immunostaining for VE-cadherin visualised continuous cell borders in WT and *Vegfr2*^*Y949F/Y949F*^ tracheal vessels, analysed using structured illumination microscopy imaging (Fig. 1g). In response to VEGFA, the WT tracheal vessels, but not *Vegfr2*^*Y949F/Y949F*^ vessels, showed a significant increase in fragmented VE-cadherin staining (Fig. 1g, quantified in h) indicative of VE-cadherin internalization and breakdown of the adherens junctions causing transient vascular leakage^13^. The difference in VE-cadherin dynamics between the genotypes was validated using endothelial cells isolated from mouse lungs. VEGFA promoted VE-cadherin junction disintegration in the WT cells but not in the *Vegfr2*^*Y949F/Y949F*^ cells, resulting in a significantly increased VE-cadherin-positive staining area in the VEGFA-treated WT cells (Fig. 1i,j).

### *Vegfr2* ^*Y949F*/Y949F^ vessel characteristics

**Figure 2:**
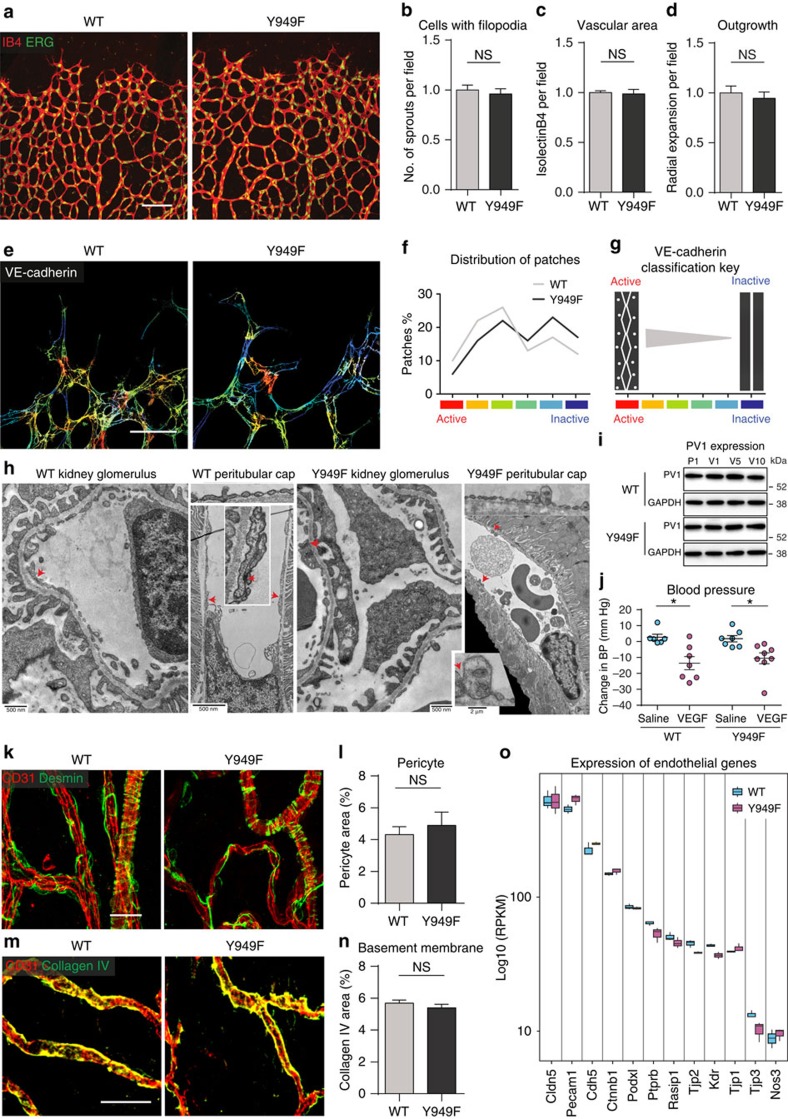
*Vegfr2*^*Y949F/Y949F*^ vessel parameters. (**a**) Isolectin-B4 (IB4, red) and ERG (green) immunostaining on WT and *Vegfr2*^*Y949F/Y949F*^ (Y949F) P6 retinal vasculature. Scale bar, 100 μm. (**b**) Tip cells numbers. *n*=3–4 retinas per genotype. (**c**) IB4-positive vessel area normalized to WT. *n*=3–4 retinas per genotype. (**d**) Radial vessel outgrowth normalized to WT. *n*=4–5 retinas per genotype. (**e**) Heatmap of VE-cadherin morphology in WT and *Vegfr2*^*Y949F/Y949F*^ (Y949F) P6 retinas. Scale bar, 50 μm. (**f**) Retinal images in **e** processed for VE-cadherin morphology, lying within the vascular mask, into ‘patches' of 100 × 100 pixels (× 63, *n*=6 per group). The VE-cadherin morphology in each patch was hand-classified according to the classification key in **g**. (**g**) Adherens junctions classification from ‘active' (red; irregular/serrated morphology with diffuse/vesicular regions) to ‘inactive' (blue; straighter morphology with less vesicular staining). (**h**) Transmission electron microscopy analysis of kidney glomerulus and peritubular capillary (cap) show normal appearance of junctions (red arrows) and fenestrae (insets) in the two genotypes. (**i**) PV1 protein immunoblot in lung lysates after injection of PBS (P) or VEGFA (V) and circulation for 1–10 min (P1; injection of PBS and 1 min circulation and so on). GAPDH; equal loading. Molecular weight markers to the right. (**j**). Blood pressure 10 min after saline or VEGFA infusion, monitored in the ascending aorta. Values (positive or negative) show diastolic blood pressure (BP) normalized to BP before VEGFA/PBS injection (70 mm Hg; NS between genotypes). *n*=6–8 mice per genotype. Student's *t*-test, *P<0.05. Two-way ANOVA: *P*(treatment)=0.0001; *P*(genotype)=0.738; *P*(interaction)=0.5321. (**k**) CD31-positive (red) tracheal endothelium and desmin-positive (green) pericytes. Scale bar; 50 μm. (**l**) Pericyte area per total area from **k**. *n*=4 mice per genotype. (**m**) CD31-positive (red) tracheal endothelium and collagen IV-positive (green) basement membrane. Scale bar; 50 μm. (**n**) Collagen IV area per total area in **m**. *n*=3 mice per genotype. (**o**) RNAseq analysis of total lung polyA^+^ RNA. Data for selected endothelial genes as log10-scaled fold-change based on the frequency of normalized expression. For *Vegfr2* (*KDR*) the Y949F mutant showed a −0.24 (DESeq2log2) fold decrease compared with WT. Data presented as mean±s.e.m. Student's *t*-test (**b**–**d**) and Wilcoxon rank test (**f**); NS, not significant. Experiments were performed three independent times or performed once with at least three independent data sets (**j**,**o**).

The *Vegfr2*^*Y949F*/Y949F^ mice showed no developmental vascular abnormality, for example, in the embryonic day 11.5 (E11.5) hindbrain (Supplementary Fig. 2a–d) and were born at the expected Mendelian ratios. There was also no apparent effect of the mutation on the development and organization of lymphatics (see Supplementary Fig. 2e for tracheal lymphatics). The postnatal retinal vasculature develops in a strictly regulated, VEGFA-dependent manner to establish the junctional barrier^14^. There was no difference in retinal vascular development between WT and *Vegfr2*^*Y949F/Y949F*^ mice as judged by the overall morphology of the vascular network, the abundance of endothelial tip cells and filopodia, and the total vascular area (Fig. 2a–d). Thus, development of the retinal vasculature does not depend on VEGFR2-Y949 signalling. However, the morphology of the VE-cadherin immunostaining differed between the two genotypes. The VE-cadherin pattern at individual junctions was evaluated using established image analysis software^15^, classifying junctions in a graded scale from ‘active'—that is, being irregular/serrated with vesicular/diffuse appearance—to ‘inactive' ones, presenting a straighter and less vesicular morphology (Fig. 2e, see Fig. 2f,g for quantification and schematic outline). By this criterion, the WT retinal vasculature displayed a significantly higher prevalence of active adherens junctions compared with the *Vegfr2*^*Y949F/Y949F*^ retinal vasculature (Fig. 2f; active junction patches was 58.9% (WT) versus 48.1% (*Vegfr2*^*Y949F/Y949F*^); *P*=0.0367, calculated using median% in one-tailed Wilcoxon rank sum test.

Still, the enhanced barrier morphology in the VEGFR2-Y949F mutant did not interfere with overall vascular morphology or physiology. The adult vascular ultramorphology of the kidney glomeruli and peritubular capillaries (Fig. 2h) and the pancreas (Supplementary Fig. 2f) was normal in the *Vegfr2*^*Y949F*/Y949F^ mouse. Moreover, expression of plasmalemma vesicle-associated protein-1 (PV1), which is required for the formation of diaphragms covering endothelial fenestrae in vessels of endocrine organs^16^ was not affected by the VEGFR2-Y949F mutation (Fig. 2i).

To exclude the possibility that vascular leakage arrest in the *Vegfr2*^*Y949F*/Y949F^ mouse was influenced by blood pressure/flow parameters, a series of *in vivo* tests were performed. The resting blood pressure in the descending aorta was monitored continuously while infusing VEGFA or phosphate-buffered saline (PBS) by tail vein injection. VEGFA, but not PBS, caused a significant drop in blood pressure as an indication of vessel dilation, in both WT and *Vegfr2*^*Y949F*/Y949F^ mice (Fig. 2j). Moreover, blood parameters including arterial gases, oxygen saturation, serum Na^+^/K^+^ levels, as well as the haematocrit/haemoglobin levels were similar in the WT and Y949F conditions (Table 1). Tail vein injection of lectin showed that the WT and *Vegfr2*^*Y949F*/Y949F^ tracheal vessels were perfused to the same extent (Supplementary Fig. 2g), supporting the notion that the leakage arrest in the mutant mouse vasculature was not due to altered circulation. Pericyte morphology (Fig. 2k,l) and basement membrane coating (Fig. 2m,n) were similar between the genotypes. Moreover, RNAseq analyses did not reveal major differences in expression levels of key endothelial transcripts between the WT and the *Vegfr2*^*Y949F*/Y949F^ condition (Fig. 2o).

### Reduced *Vegfr2* ^Y949F/Y949F^ glioblastoma vessel leakage

**Figure 3:**
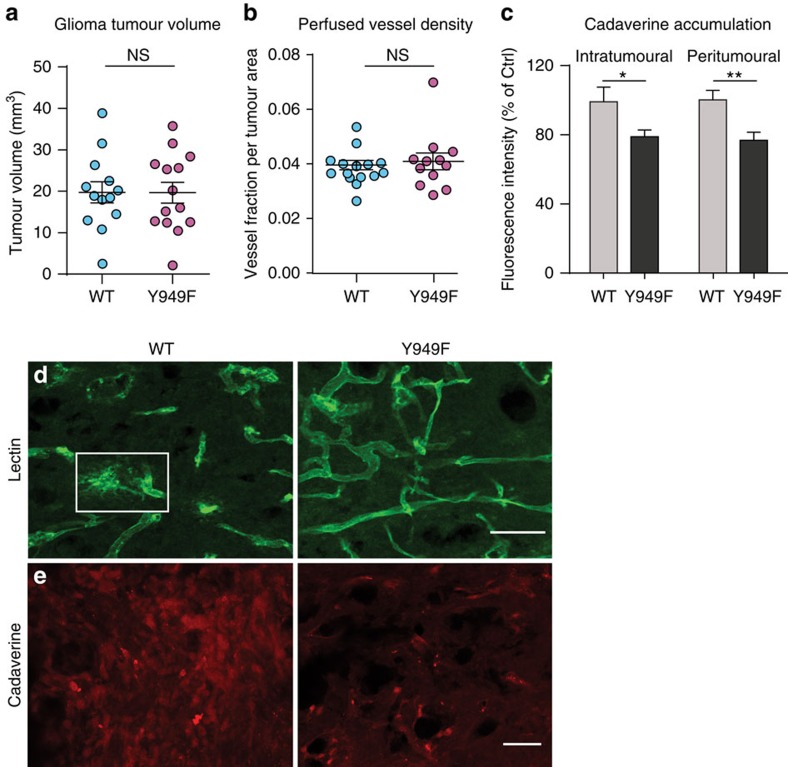
Reduced glioblastoma permeability in *Vegfr2*^Y949F/Y949F^ mice. (**a**) GL261 glioma volumes at D18, estimated by measuring all tumour-containing sections in series throughout the brain of WT and *Vegfr2*^*Y949F/Y949F*^ (Y949F) mice. *n*=13–14 mice per genotype. (**b**) Lectin-positive, perfused vessel density (area fraction per tumour area) at D18. *n*=12–14 mice per genotype. (**c**) Cadaverine-555 fluorescence intensity units estimated within the tumour (intratumoural) and at a 0–500-μm rim around the tumour border (peritumoural), Y949F normalized to WT. *n*=8–11 mice per genotype. (**d**) GL261 lectin-perfused vessels (lectin in green). Boxed area, typical point of leakage/disrupted barrier in the WT. (**e**) Cadaverine-555 (red) extravasation in WT and Y949F GL261 gliomas. Scale bar, 100 μm (**d**) and 50 μm (**e**). Data are presented as mean±s.e.m. Student's *t*-test, *P<0.05, **P<0.01. Experiments were performed three independent times. NS, not significant.

The integrity of the central nervous system vasculature is tightly controlled by the blood–brain barrier, in which endothelial junctions are particularly tight^17^. We asked whether VEGFA-regulated vascular leakage has an impact on the growth and vascular permeability of brain tumours. The GL261 glioma model has many features reminiscent of human glioma, including abnormal vasculature, high VEGFA production and oedema^18^. GL261 glioma cells were injected in the right medial striatum of WT and *Vegfr2*^*Y949F/Y949F*^ mice. There was no effect on tumour growth at any examined time point after cell inoculation (day 7 (D7), D14, D18 and D21; Fig. 3a shows tumour volume at D18). There was also no difference in the extent of perfused vessel density (Fig. 3b). However, leakage of tail vein-injected cadaverine-Alexa-555, a small tracer that normally does not cross the blood–brain barrier^19^, was reduced in the *Vegfr2*^*Y949F/Y949F*^ gliomas compared with WT at D18, both intratumourally and in the tumour periphery (0–500 μm peritumourally; Fig. 3c). The glioma vasculature in the WT was more disorganized than in the *Vegfr2*^*Y949F/Y949F*^ (Fig. 3d). Hotspots of fluorescent lectin leaking from perfused vessels (Fig. 3d, boxed area) correlated with high tumour uptake of Cadaverine-555 (Fig. 3e) in the WT compared with the mutant.

### *RIP1-TAg2:Vegfr2* ^*Y949F/Y949F*^ leakage and metastatic spread

**Figure 4:**
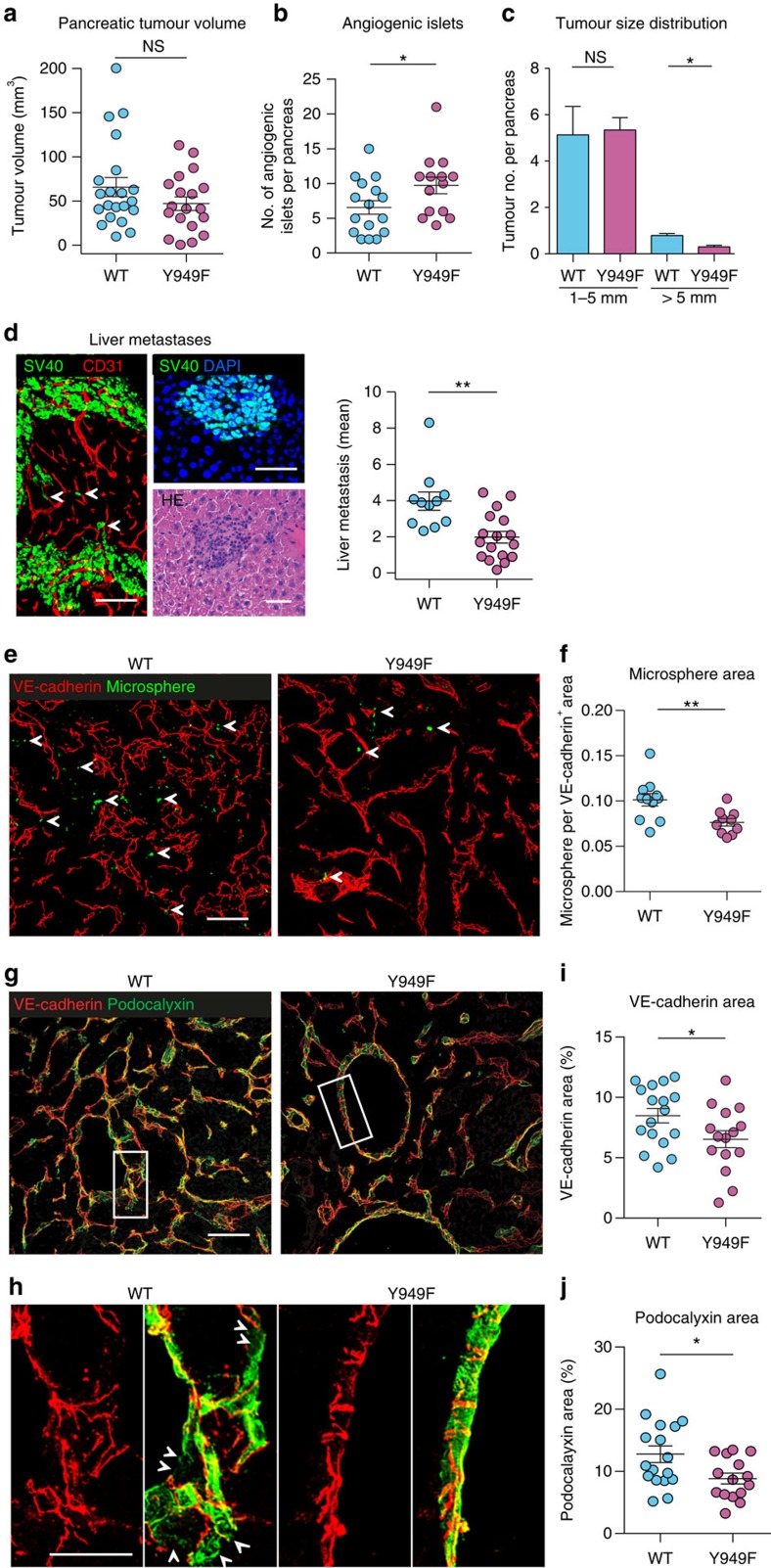
Reduced RIP1-TAg2 vascular leakage and metastatic spread in *Vegfr2*^*Y949F/Y949F*^. (**a**) RIP1-TAg2 WT and *Vegfr2*^*Y949F/Y949F*^ insulinoma tumour volumes at week 14. *n*=19–21 mice per genotype. (**b**) Angiogenic islet numbers at week 14. *n*=14–16 mice per genotype. (**c**) Tumour size distribution (1–5 and >5 mm diameter), week 14. *n*=17–19 mice per genotype. (**d**) Tumour cells (SV40 T antigen^+^; green) and vessels (CD31^+^; red) in RIP1-TAg2 insulinomas (left), Arrows; individual tumour cells associated with vessels. Scale bar, 100 μm. SV40 T antigen^+^ (green; upper) and haematoxylin-eosin (HE; lower) metastatic liver lesion (right). Scale bars, 50 μm. Number of liver metastases; mean per section, 30 sections per liver. *n*=11–17 mice per genotype. (**e**) Leakage of 30-nm microspheres (green; arrows) from VE-cadherin^+^ tumour vessels (red). Scale bar, 50 μm. (**f**) Microspheres area per VE-cadherin area from **e**. *n*=10–11 tumours per genotype. (**g**,**h**) VE-cadherin^+^ (red) and podocalyxin^+^ (green) RIP1-TAg2 tumour vasculature. Lower panels, magnification of boxed areas in upper panels. Arrows indicate typical disorganized podocalyxin in WT tumour vasculature. Scale bars, 50 μm (**g**), 5 μm (**h**). (**i**) VE-cadherin area, % of total area from **g**. *n*=15–17 tumours per genotype. (**j**) Podocalyxin area, % of total area from **g**. *n*=15–17 tumours per genotype. Data shown as mean±s.e.m. Student's *t*-test, *P<0.05, **P<0.01, NS, not significant. Data are pooled from three independent studies.

The consequence of the stabilized endothelial junctions in *Vegfr2*^*Y949F/Y949F*^ mice during tumour progression was further explored in the *RIP1-TAg2* model. β-cells in the islets of Langerhans in RIP1-TAg2 mice express SV40 T antigen controlled by the rat insulin promoter, giving rise to multiple spontaneous pancreatic neuroendocrine tumours^20^ that seed metastases in the liver^21^. While there was no difference in the total tumour volume between the *RIP1-TAg2:WT* and *RIP1-TAg2:Vegfr2*^*Y949F/Y949F*^ genotypes (Fig. 4a), the number of angiogenic islets, which are small lesions representing an early stage in tumour development, was significantly increased in *RIP1-TAg2:Vegfr2*^*Y949F/Y949F*^ mice (Fig. 4b). Conversely, the *RIP1-TAg2:Vegfr2*^*Y949F/Y949F*^ presented with fewer large (>5 mm) neoplastic islet tumours (Fig. 4c). Tumour cells were found in close proximity to the vasculature in the tumour periphery suggesting metastatic spread of the insulinoma (Fig. 4d). Examination of livers showed that the number of SV40 T-positive liver metastases in the *RIP1-TAg2:WT* was significantly higher than in *RIP1-TAg2:Vegfr2*^*Y949F/Y949F*^ mice at week 14 (Fig. 4d). We hypothesized that reduced metastatic spread in *RIP1-TAg2:Vegfr2*^*Y949F/Y949F*^ mice could correlate with reduced vascular leakage. Tail vein-injected microspheres leaked from tumour vessels to a higher extent in *RIP1-TAg2:WT* than in *RIP1-TAg2:Vegfr2*^*Y949F/Y949F*^ tumours (Fig. 4e,f). In the *RIP1-TAg2:Vegfr2*^*Y949F/Y949F*^ tumour vasculature, VE-cadherin was preferentially localized at endothelial junctions and the VE-cadherin area was lower than in the WT (Fig. 4g–i). Moreover, staining for the sialomucin podocalyxin decorated the luminal endothelial surface in the *RIP1-TAg2:Vegfr2*^*Y949F/Y949F*^ tumour vasculature while *RIP1-TAg2:WT* tumours showed a chaotic podocalyxin staining pattern suggestive of altered apical–basal (luminal–abluminal) endothelial polarity (Fig. 4g,h,j) and therefore, disturbed vessel organization. The extent of vessel perfusion, pericyte coating and inflammatory cell infiltration (that is, CD45, F4/80 and macrophage mannose receptor (MMR)-positive cells did, however, not differ between *RIP1-TAg2:WT* and *RIP1-TAg2:Vegfr2*^*Y949F/Y949F*^ tumours at week 14 (Supplementary Fig. 3a–e).

### B16F10 vessel leakage and metastatic spread

**Figure 5:**
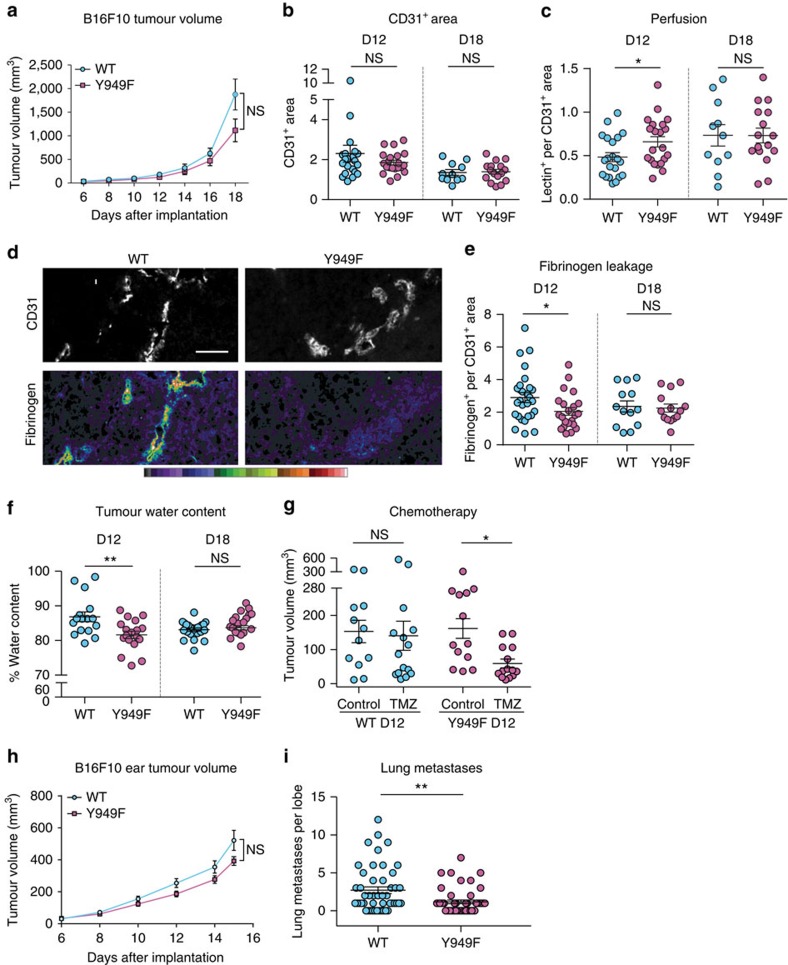
Reduced B16F10 vascular leakage and metastatic spread in *Vegfr2*^*Y949F/Y949F*^. (**a**) Subcutaneous B16F10 tumour volumes for WT (blue) and *Vegfr2*^*Y949F/Y949F*^ (Y949F; magenta) mice at different days after inoculation. *n*=14–15 mice per genotype. Repeated measures ANOVA: *P*(time)<0.0001; *P*(genotype)=NS. (**b**) CD31^+^ area per field in B16F10 tumours from WT and Y949F mice. *n*=21 mice per genotype at D12 and 11–16 mice per genotype at D18. (**c**) Area of tomato lectin-perfused vessels normalized to CD31^+^ area. *n*=21 mice per genotype, D12; and 11–16 mice per genotype at D18. *P<0.05. (**d**) Tumour vasculature (upper panels; CD31^+^ vessels; white) and perivascular fibrinogen deposition (lower panels, heatmap from red, high, to blue, low) in B16F10 tumours from WT and *Vegfr2*^*Y949F/Y949F*^ mice at D12. Scale bars, 50 μm. (**e**) Fibrinogen^+^ area normalized to CD31^+^ area. *n*=22–27 mice per genotype at D12 and 13–14 mice per genotype at D18. Student's *t*-test, *P*=0.0310. (**f**) Oedema in B16F10 tumours estimated by weighing tumours before and after drying. Data show (wet weight−dry weight)/wet weight in % at D12 and D18. *n*=16-18 mice per genotype at D12 and 19–24 mice per genotype at D18. Student's *t*-test, *P*=0.0385. (**g**) Tumour volumes at D12 of TMZ or vehicle (dimethylsulfoxide)-treated mice with B16F10 tumours, receiving treatment between D4 and D8 after inoculation. *n*=12–15 mice per group. Kruskal–Wallis test, *P*=0.0289. (**h**) B16F10 tumour volumes in mouse ear. *n*=12–13 mice per genotype. Repeated measures ANOVA: *P*(time)<0.0001; *P*(genotype)=NS. (**i**) Spontaneous B16F10 lung metastasis spreading to the lungs from primary B16F10 tumours in the ear. dsred-B16F10 metastases in four longitudinal sections of each left main lung lobe were counted. *n*=12 mice per genotype. Mann–Whitney *U*, *P*=0.0028. Data shown as mean±s.e.m. Dashed lines in **b**,**c**,**e** and **f** indicate that data sets from D12 and D18 were not compared statistically. *P<0.05, **P<0.01, NS, not significant. Experiments were performed three independent times (**a**–**e**,**h**–**j**) or two times (**f**,**g**), and data were pooled.

To further test the effect of the VEGFR2-Y949F mutation on vascular integrity in cancer, we chose to study subcutaneous B16F10 melanoma^22^. B16F10 tumour growth in the back skin was monitored continuously showing similar growth curves for the two genotypes (Fig. 5a). To monitor dynamic changes in tumour and vascular parameters, tumours were collected at two different time points after inoculation, at D12 (average tumour volume 200 mm^3^) and at D18 (average tumour volume 1.5 cm^3^) from WT and *Vegfr2*^*Y949F/Y949F*^ mice. Vascular area (Fig. 5b) and pericyte density (Supplementary Fig. 3f) were similar in WT and *Vegfr2*^*Y949F*/Y949F^ B16F10 tumours at D12 and D18. Vessel functionality as assessed by lectin perfusion was more efficient in the *Vegfr2*^*Y949F/Y949F*^ tumour vasculature at D12 (Fig. 5c). At D18, vessel perfusion had improved in the WT and was similar to that of *Vegfr2*^*Y949F*/Y949F^ tumours. In spite of the improved perfusion, necrosis in the tumour was similar in the two genotypes (Supplementary Fig. 3g). Importantly, there were no differences in infiltration of F4/80^+^, CD45^+^ and MMR-positive inflammatory leukocytes in the tumours at D12 (Supplementary Fig. 3h–j).

Vessel leakage in B16F10 tumours, determined by estimating the deposition of perivascular fibrinogen (Fig. 5d; quantified in e), was significantly lower in *Vegfr2*^*Y949F/Y949F*^ tumours compared with WT tumours at D12. At D18, fibrinogen deposition was reduced in the WT tumours and similar between the two genotypes. Vascular leakage in tumours leads to oedema, which was estimated by weighing tumours before and after drying. The water content of *Vegfr2*^*Y949F/Y949F*^ tumours was significantly lower than in WT tumours at D12 (Fig. 5f) but not at D18. Altogether, these data indicate that the block in VEGFR2 pY949 signalling constrained vascular leakage and oedema in the tumour up to a critical point in tumour growth, occurring around D12. Thereafter, leakage and oedema were reduced irrespective of genotype, possibly due to an overall dysfunctional tumour circulation.

To test the effect of chemotherapy on tumours exhibiting different extents of vessel leakage and oedema, B16F10 tumour-bearing mice were treated with a subtherapeutic dose of temozolomide (TMZ) or vehicle in one cycle for 5 consecutive days. Tumour growth in WT mice was resistant to the TMZ treatment at the given dose. In contrast, TMZ-treated *Vegfr2*^*Y949F/Y949F*^ mice had smaller tumours than WT at D12 (Fig. 5g) but not at D18 (Supplementary Fig. 4a). Thus, the VEGFA-resistant vessel barrier phenotype in the *Vegfr2*^*Y949F/Y949F*^ tumours correlated with an enhanced drug effect at D12, probably due to the lower oedema, and hence a lower interstitial tumour pressure, at this stage.

To study B16F10 metastasis, dsRed-B16F10 cells were inoculated intradermally in the ear. Tumour growth rate is constrained in the ear dermis, which we found correlated with an increased propensity to metastasize compared with when tumours grow unconstrained in the back skin. The primary tumour growth rate was similar between *Vegfr2*^*Y949F/Y949F*^ and WT mice (Fig. 5h). Metastatic spread from the primary dsRed-B16F10 tumour to the lungs was however significantly reduced in the *Vegfr2*^*Y949F/Y949F*^ mice (Fig. 5i). There was no correlation between the end point weight of the primary tumour and the number of metastatic foci (Supplementary Fig. 4b). We further tested the capacity of tail vein-injected dsRed-B16F10 tumour cells to colonize lungs and found no difference in lodging frequency between the genotypes (Supplementary Fig. 4c).

### *In vivo* signalling in the *Vegfr2* ^*Y949F/Y949F*^ endothelium

To decipher signalling regulating VEGFA-induced vascular leakage *in vivo*, mice were subjected to tail vein injection of VEGFA or vehicle, and lungs were collected after different time periods of circulation (1–60 min). Lungs were chosen since they consist at least 50% of endothelial cells. Moreover, we used the two-tracer technique^23^ to show that WT lung endothelial cells respond to VEGFA with increased transvascular leakage of iodinated albumin *in vivo*. The leakage rate in WT mice increased 2.5-fold after VEGFA injection in the tail vein, compared with vehicle (flow rate in response to VEGFA, 0.859±0.114 versus vehicle, 0.334±0.126 μl min^−1^ g^−1^; s.e.m., *P*=0.0278, *n*=3–4 mice per condition).

**Figure 6:**
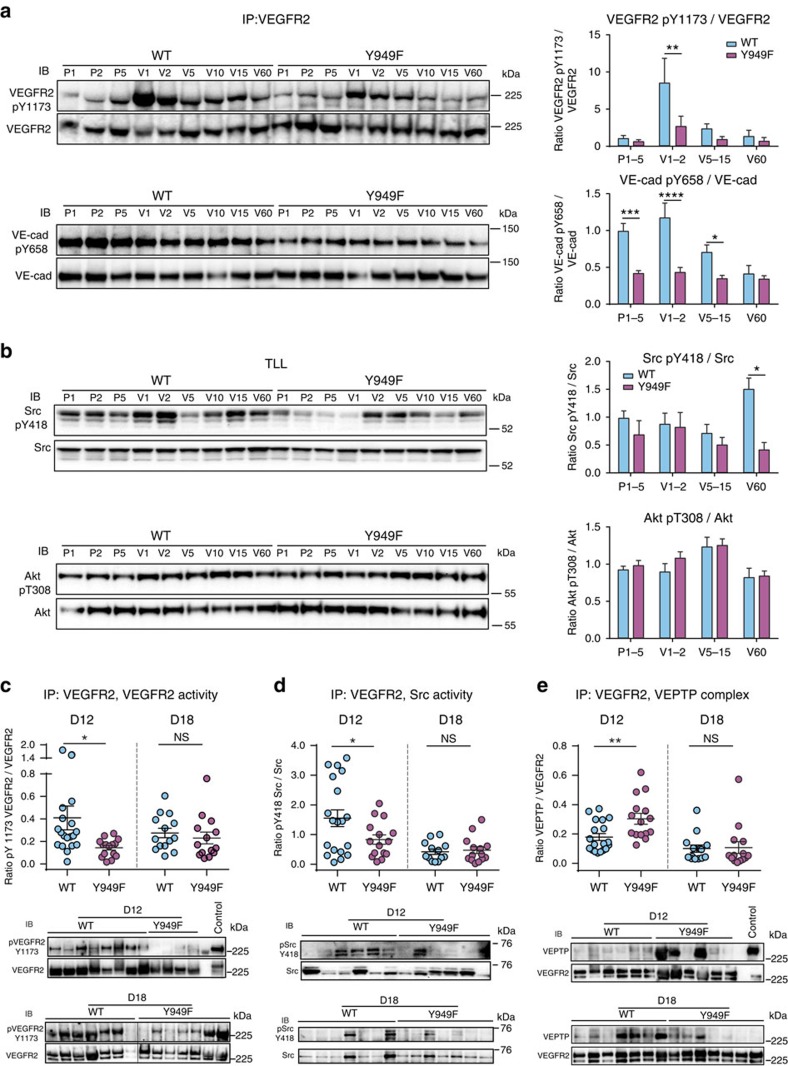
VEGFA-induced signalling in *Vegfr2*^*Y949F/Y949F*^. (**a**) VEGFR2 immunoprecipitation (IP) and immunoblotting (IB) for pY1173VEGFR2, VEGFR2, pY658VE-cadherin and VE-cadherin from lung lysates after tail vein injection of PBS (P) or VEGFA (V) in WT and *Vegfr2*^*Y949F/Y949F*^ (Y949F) mice, followed by circulation for different time periods (P1, P2, P5: PBS injection and circulation for 1, 2 or 5 min; V1, V2, V5, V10, V15, V60: VEGFA injection and circulation for 1, 2, 5, 10, 15 or 60 min). Quantification shown to the right; *n*=4. Groups of early (V1–V2), intermediate (V5, V10, V15) and late (V60) time points were quantified. Phosphoform/total protein band densities were normalized to the mean of all PBS samples. Two-way ANOVA, upper (p1173VEGFR2): *P*(genotype)=0.032, *P*(time)=0.0009; lower (VE-cadherin): *P*(genotype) <0.0001, *P*(time)=0.0049. (**b**) VEGFA-induced accumulation of pY418 Src (upper) and pT308 Akt (lower) in total lung lysates (TLL) from *Vegfr2*^*Y949F/Y949F*^ (Y949F) mice. Quantification as in **a**, relative to the unphosphorylated c-Src and Akt pools and normalized to mean PBS values. Two-way ANOVA, upper (Src): *P*(genotype)=0.0137, *P*(time)=NS; lower (Akt): *P*(genotype)=NS, *P*(time)=0.0013. (**c**) Quantification (top) of IB (bottom) for pY1173VEGFR2 and VEGFR2 in VEGFR2 immunoprecipitates (IP) from B16F10 tumours at D12 and D18 after inoculation of WT and *Vegfr2*^*Y949F/Y949F*^ (Y949F) mice. *n*=19/WT D12, 14/WT D18, 15/Y949F D12 and 14/Y949F D18. (**d**) Quantification (top) of IB (bottom) for pY418Src and c-Src in VEGFR2 immunoprecipitates (IP) from tumour lysates as in **c**. *n*=19/WT D12, 13/WT D18, 14/Y949F D12 and 14/Y949F D18. (**e**) Quantification (top) of IB (bottom) for VEPTP and VEGFR2 on VEGFR2 immunoprecipitates (IP) from tumour lysates as in **c**. *n*=19/WT D12, 14/WT D18, 15/Y949F D12 and 14/Y949F D18 tumours. Data shown as mean±s.e.m. Dashed lines in **c**–**e** indicate that data sets from D12 and D18 were not compared statistically. In **a**,**b** two-way ANOVA, with Sidak's *post hoc* test; in **c**–**e** Student's *t*-test. *P<0.05, **P<0.01, ***P<0.0001, ****P<0.00001. Experiments were performed four independent times.

VEGFA induced a very rapid and strong phosphorylation of VEGFR2 at Y1173 in WT and to a lower relative level in the *Vegfr2*^*Y949F/Y949F*^ lung endothelium (Fig. 6a). Phosphorylation at Y949 was also high at 1 min after VEGFA injection in the WT, whereas it was absent in the *Vegfr2*^*Y949F/Y949F*^ vasculature (Supplementary Fig. 5a). The lower pY1173 accumulation in the *Vegfr2*^*Y949F/Y949F*^ could be due to the more extensive complex formation with the vascular endothelial protein tyrosine phosphatase (VEPTP). As shown in Supplementary Fig. 5b, there was a significantly higher extent of VEPTP/VEGFR2 complex formation in the *Vegfr2*^*Y949F/Y949F*^ immunoprecipitates compared with WT VEGFR2 (two-way analysis of variance (ANOVA), genotype effect: *P*=0.0037). VEPTP has been shown to restrict VEGFR2 tyrosine phosphorylation^24^.

Several signalling pathways regulate vascular permeability and leakage, including VEGFA-induced c-Src signalling^8^,^25^ and histamine-induced phosphoinositide 3 kinase/Akt signalling^26^, which both induce phosphorylation of VE-cadherin and VE-cadherin internalization. We examined induction of pY658 VE-cadherin by immunoblotting of VEGFR2 immunoprecipitates from mouse lungs. We choose to study VE-cadherin in complex with VEGFR2 to monitor direct communication between the two. In the WT, the levels of pY658 VE-cadherin were already high in the PBS-injected mice in agreement with previous reports^27^, and remained high initially after injection of VEGFA, followed by a gradual decrease possibly due to the action of phosphatases, or to internalization and degradation of VE-cadherin (Fig. 6a). In the *Vegfr2*^*Y949F*/Y949F^-derived samples, the pVE-cadherin level was low in the PBS condition and there was no induction with VEGFA injection (Fig. 6a).

Tyrosine phosphorylation of c-Src was difficult to detect in the VEGFR2 immunoprecipitates from lung lysate and was therefore examined in total lung lysates (Fig. 6b). In the WT samples, we detected an increased accumulation of pY418 c-Src only at the late time point, in agreement with previous data where c-Src activation was detected in lung lysates 20 min after tail vein injection of VEGFA^8^. In the *Vegfr2*^*Y949F/Y949F*^ mouse lungs, tyrosine phosphorylation of c-Src Y418 increased with VEGFA during the early phase but did not accumulate in the later phase of signalling (Fig. 6b, upper). Thus, the profiles of VEGFA-induced, endothelial c-Src phosphorylation differed between the WT and *Vegfr2*^*Y949F/Y949F*^ mice. On the other hand, pT308AKT, examined in total lung lysates, accumulated with relatively slow and similar kinetics in the WT and *Vegfr2*^*Y949F/Y949F*^ lungs in response to VEGFA injection (Fig. 6b, lower). These data indicate that the VEGFR2-Y949F mutation interfered with c-Src activation and pathways downstream of c-Src, including VE-cadherin phosphorylation at Y658.

VEGFA signalling in B16F10 tumours was examined to understand how leakage was regulated in a tumour context. Immunoblotting of lysates from individual tumours showed that the pY1173/VEGFR2 ratio was significantly higher at D12 in tumours from WT mice compared with *Vegfr2*^*Y949F/Y949F*^ mice (Fig. 6c; lower part shows representative immunoblot), correlating with the VEGFA signalling data from lung lysates (Fig. 6a). At D18, the pVEGFR2/VEGFR2 ratios were similar between the genotypes. The results on the pVEGFR2/VEGFR2 levels are likely to reflect the status in the tumour vasculature, as B16F10 cells do not express VEGFR2 (ref. 28). Immunoblotting for pY418Src in the VEGFR2 immunoprecipitates from tumour lysates showed a significantly higher pSrc/Src ratio in the D12 tumours from WT mice compared with *Vegfr2*^*Y949F/Y949F*^ mice (Fig. 6d; lower part shows representative blot). At D18, there was no difference between the genotypes in the extent of pSrc accumulation.

The restriction in VEGFR2 signalling in tumours from the *Vegfr2*^*Y949F/Y949F*^ mice at D12 may involve dephosphorylation of VEGFR2 by VEPTP, as we found increased levels of VEGFR2/VEPTP complexes in the Y949F condition (Fig. 6e; lower part shows representative blot). VEPTP expression levels in D12 tumours did not differ between the genotypes (Supplementary Fig. 5c). At D18, VEGFR2/VEPTP complex formation was similar between WT and *Vegfr2*^*Y949F/Y949F*^-derived tumours (Fig. 6e). For uncropped versions of all blots presented in this study, see Supplementary Figs 6–13.

**Figure 7:**
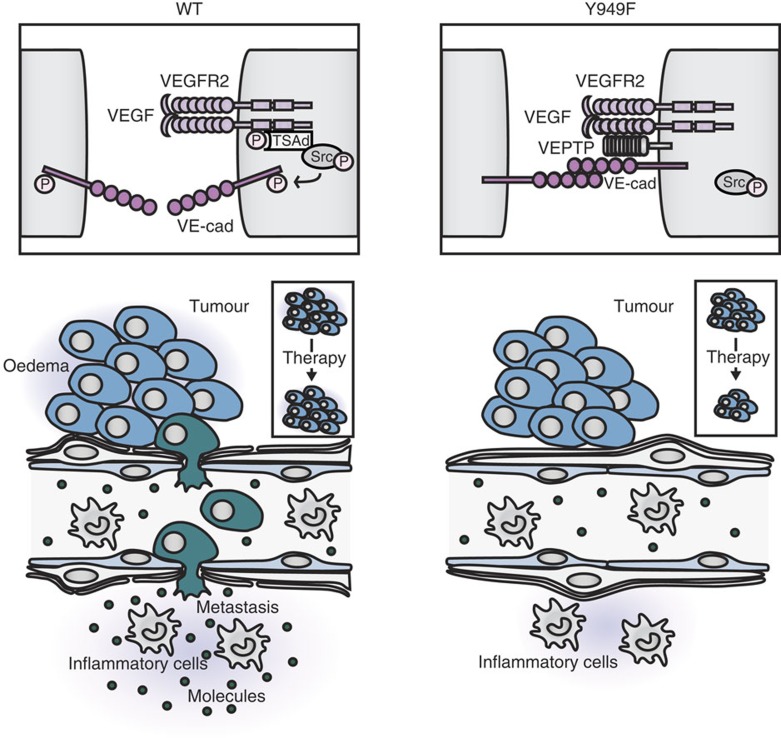
Schematic outline of Y949 signalling in VEGFA-regulated vascular permeability. WT and *Vegfr2*^*Y949F/Y949F*^ (Y949F) endothelial junctions are shown (upper). In the WT, the junction is active, that is, VE-cadherin is not engaged in homophilic interaction, and VEGFA signalling results in c-Src activation at junctions. In the Y949F mutant, VEGFR2 remains in complex with VEPTP and VE-cadherin, promoting junctional quiescence. c-Src may be activated but not at junctions. Lower schematics shows that junctional activation in the WT results in extravasation of cells and molecules, causing tissue oedema as well as metastatic spread via tumour cell intravasation into the blood stream (cells indicated in green). In the Y949F mutant, extravasation and oedema are blocked and metastatic spread is suppressed, while inflammatory cell extravasation is unaffected.

We conclude that VEGFA-induced leakage (see Fig. 7 for a schematic outline of VEGFA signalling regulating vascular permeability/leakage) can be restricted during a window of time of B16F10 tumour growth, by suppressing the pY949 pathway. Moreover, the data indicate that VEGFA signalling is dynamically regulated by VEPTP in solid tumours.

## Discussion

Here we show that a specific arrest in VEGFA-induced vascular permeability leading to attenuation of molecular extravasation in organs such as the trachea and skin is compatible with normal vascular development, morphology and function. The *Vegfr2*^*Y949F/Y949F*^ mouse displayed reduced vascular leakage in several orthotopic tumour models and a significant reduction in metastatic spread of insulinoma and melanoma (see Fig. 7 for a schematic summary of the results). The enhanced vascular barrier in the *Vegfr2*^*Y949F/Y949F*^ mouse led to reduced tumour oedema, allowing for increased chemotherapeutic efficacy. While our data strongly indicate that signalling via pY949-VEGFR2 regulates c-Src activation and VE-cadherin phosphorylation and therefore adherens junction integrity, we do not rule out the involvement of transendothelial pores/vesiculovacuolar organelles in VEGFA-induced vascular permeability^29^.

Induced vascular permeability, also denoted vascular hyperpermeability or vascular leakage, may be acute or chronic^30^. The best studied factors regulating vascular leakage include VEGFA and inflammatory cytokines such as histamine and bradykinin^31^. An implication from the current study is that VEGFA dominates over inflammatory cytokines in regulating molecular extravasation, in the models studied here. VEGFA was originally identified as a vascular permeability factor^30^, but the physiological role of vascular permeability regulated by VEGFA/VPF has remained unclear. Dvorak and colleagues implicated vascular permeability in the formation of a provisional matrix for the growth of new vessels^32^. Our data do not support an essential role for the VEGFA-driven pathway regulated by VEGFR2 pY949 signalling, in angiogenesis.

Cheresh and coworkers have shown the critical role for c-Src-pathways in regulation of VEGFA-induced vascular permeability^25^. Our work extends these data by implicating pY949-VEGFR2/TSAd in regulation of c-Src pY418, VE-cadherin pY658 and the stability of adherens junctions. VE-cadherin is constitutively tyrosine phosphorylated in postcapillary venules compared with arteries/arterioles and regulated through flow-induced activation of c-Src, as judged from immunofluorescent staining of different capillary beds^27^. In this study, we noted a marked reduction in VE-cadherin Y658 phosphorylation in the *Vegfr2*^*Y949F*/Y949F^ lung lysates compared with WT, in agreement with the altered c-Src phosphorylation profile (Fig. 6a,b) and with the persistence of adherens junctions in response to VEGFA, *in vivo* and *in vitro* (Fig. 1g–j). However, the complex biology of c-Src precludes a direct cause–effect relationship between c-Src pY418 phosphorylation and VE-cadherin/adherens junction stability. We have previously shown that pY418 Src accumulates with relatively slow kinetics *in vivo* and that it translocates to junctions in complex with the adaptor molecule TSAd^8^. It is possible that the dynamics of c-Src activation differs between different subcellular compartments such as junctions and focal adhesions. Thus, at focal adhesions, c-Src activity is induced as a consequence of cell adhesion-dependent focal adhesion kinase activation, which also affects vascular permeability most likely by orchestrating cell shape^33^. In agreement with the data presented here, focal adhesion kinase (*Ptk2*) gene knockout decreases tumour spread^34^. Owing to technical constraints imposed by the quality of commercial c-Src antibodies, we have not been able to distinguish different subcellular pY418 Src pools *in vivo*. However, accumulation of pY418 Src in relation to the total c-Src pool was decreased both in *Vegfr2*^*Y949F*/Y949F^ lung endothelial cells and in B16F10 tumours growing in mutant mice.

VEGFA-induced accumulation of pT308 Akt was similar in the WT and mutant Y949F VEGFR2 conditions. Akt plays an important role in vascular permeability induced by inflammatory cytokines^26^. Akt is critical for the activation of endothelial NO synthase (eNOS) *in vivo*, by phosphorylation on Ser1176 (ref. 35), resulting in increased NO production. NO, a potent regulator of the vascular tone, mediates vasodilation by stimulating soluble guanylyl cyclase and increasing cyclic GMP in smooth muscle cells^36^. In this study, we recorded transiently reduced blood pressure indicative of vasodilation in response to VEGFA in the descending aorta (Fig. 2j) in both WT and *Vegfr2*^*Y949F/Y949F*^ mice. This result together with the full activation of Akt, judged from the pattern of pT308 Akt accumulation in the mutant, indicate that the NO pathway is intact in the *Vegfr2*^*Y949F/Y949F*^ mouse. eNOS has previously been implicated in tumour vascular permeability, and inhibition of eNOS activity by a small caveolin-derived peptide (cavtratin) leads to suppressed tumour vascular permeability and reduced primary tumour growth without affecting blood flow and angiogenesis^37^. In contrast, while metastatic spread was suppressed in the *Vegfr2*^*Y949F/Y949F*^ mouse, primary tumour growth was only modestly affected.

We noted reduced phosphorylation of VEGFR2 at phosphosite Y1173 in the *Vegfr2*^*Y949F/Y949F*^ lungs and in D12 B16F10 melanoma. Mutation of the human tyrosine residue, Y951 (corresponding to mouse Y949), in a purified recombinant intracellular VEGFR2 domain interferes with protein folding^38^. Whether protein folding is affected also in the full-length VEGFR2 remains to be shown. We noted that the level of phosphorylation of the full-length mouse VEGFR2-Y949F mutant relative to WT VEGFR2 varied during tumour progression. Thus, in B16F10 tumours, the relative level of pY1173 in WT VEGFR2 was higher than for VEGFR2-Y949F at an early stage, whereas the levels were similar at later stages of tumour growth. We propose that the reduced VEGFR2 phosphorylation in the *Vegfr2*^*Y949F/Y949F*^ lungs in D12 B16F10 tumours at least in part was a consequence of sustained VEGFR2/VEPTP complex formation. VEPTP is a receptor-type phosphotyrosine phosphatase specifically expressed in endothelial cells and known to have VE-cadherin and Tie2 as direct substrates^39^. VEGFR2 is an indirect substrate for VEPTP, resulting in dephosphorylation of VEGFR2 in complex with Tie2 (ref. 24). Here we confirm and extend the data on the important role of VEPTP as an *in vivo* regulator of VEGFR2 activity. VEGFR2/VEPTP complex formation correlated with VEGFR2 pY1173 levels and the extent of oedema. Thus, B16F10 melanomas in WT mice showed low VEGFR2/VEPTP complex formation, high p1173 VEGFR2 levels and prominent oedema. In contrast, melanomas in the *Vegfr2*^*Y949F/Y949F*^ mice displayed high VEGFR2/VEPTP complex formation, low p1173 pVEGFR2 levels and reduced oedema. With time, VEPTP/VEGFR2 complex formation decreased in tumours from both WT and *Vegfr2*^*Y949F/Y949F*^ mice. Still, the VEGFR2 and c-Src activities were suppressed in the late stage of tumour growth in WT mice, showing a restriction in VEGFA signalling independent of VEPTP. The gradual progression towards lower VEGFR2 phosphotyrosine levels over time is in agreement with models for resistance towards anti-VEGFA neutralizing therapy in cancer^40^.

The extent of tumour oedema correlates to the blood supply, the volume of blood entering the tumour and the lymphatic drainage^41^. Tumour blood supply decreases as the tumour mass increases^42^, most likely due to a progressive deterioration of the tumour vasculature with eventually fewer functional vessels. Tumours in *Vegfr2*^*Y949F*/Y949F^ mice were less oedematous, which we attribute to the higher vessel integrity in the mutant. Excess and persistent extravasation increases the interstitial pressure and impairs delivery of therapeutics to the tumour tissue^4^. In agreement, the B16F10 tumour-bearing *Vegfr2*^*Y949F/Y949F*^ mice were more responsive to treatment with chemotherapy. Complete sealing of junctions to VEGFA, as well as to inflammatory cytokines, as a result of expression of a VE-cadherin/α-catenin fusion protein, leads to a partial reduction in inflammatory cell extravasation^43^. In contrast, restricting VEGFA-induced permeability alone as in the *Vegfr2*^*Y949F/Y949F*^ genotype, failed to suppress inflammatory cell infiltration in RIP1-TAg2 insulinomas (Supplementary Fig. 3c–e) and B16F10 melanomas (Supplementary Fig. 3h–j). While we do not exclude that acute inflammation or other aspects of inflammation not studied here are affected by the VEGFR2 mutation, our data suggest that there is no direct relationship between the capacity to spread metastasis and primary tumour inflammation. Moreover, lodging of circulating tumour cells was unaffected by the mutation (Supplementary Fig. 4b) suggesting that the stable *Vegfr2*^*Y949F*/Y949F^ junctions may restrict the ability of tumour cells to enter but not exit the circulation. The caveats of the experimental metastasis model^44^ (that is, injection of tumour cells in the tail vein followed by lodging of tumour cells in the lung) compared with spontaneous metastasis, limits conclusions however.

Possibly, factor(s) other than VEGFA may contribute to extravasation of B16F10 cells in the lung; indeed, the *Vegfr2*^*Y949F*/Y949F^ endothelium retains sensitivity to other permeability-inducing factors than VEGFA such as histamine. It is pertinent to ask whether the reduced metastatic spread in the *Vegfr2*^*Y949F*/Y949F^ tumours can be attributed directly to the VEGFA-resistant Y949F junctions, that is, do tumour cells intravasate into the circulation via junctions? Alternatively, were other parameters, established as a consequence of the nonleaky junctions and the reduced oedema, more important? As we did not record significant changes compatible with vascular normalization^4^, that is, in vascular density, pericyte coating, necrosis or inflammation in the B16F10 melanomas from WT and mutant mice, we propose that the *Vegfr2*^*Y949F*/Y949F^ phenotype primarily depends on the VEGFA-resistant junctions.

In conclusion, our data suggest that specifically suppressing VEGFA-induced vascular permeability without attenuation of vital aspects of VEGFA biology, for example, endothelial survival and neoangiogenesis, may offer an attractive strategy in future drug development to reduce oedema, to improve therapeutic efficacy and to suppress metastatic spread in cancer.

## Methods

### *Vegfr2* ^*Y949F/Y949F*^ mouse model

A mouse model with knock-in of phenylalanine (F) to replace the tyrosine (Y) at position 949 (Y951 in the human) of VEGFR2 (designated *Vegfr2*^*Y949F/Y949F*^) in a mixed 129S6/C57BL/6 was created using VelociGene technology (Regeneron Pharmaceuticals, New York, USA)^11^. The *Vegfr2*^*Y949F/Y949F*^ mouse was backcrossed onto the C57BL/6 background for more than 10 generations and maintained through crossing of *Vegfr2*^*Y949F/Y949F*^ heterozygotes. All data shown are based on comparison of *Vegfr2*^*Y949F/Y949F*^ and WT littermates obtained from heterozygote crossings in experiments repeated at least three times. The Uppsala University board of animal experimentation approved all animal work; efforts were done to minimize suffering as supervised by the University veterinarian. Mice were anaesthetized with ketamine/xylazine, avertin or isoflurane (Isoba, 3% induction and 1.5% maintenance).

### Statistical analysis

For parametric comparison, Student's *t*-test was performed to compare means between two groups. One-way ANOVAs were performed to compare >2 treatment groups and two-way ANOVA was used when two factors were involved, for example, treatment and genotype. Repeated measures ANOVA was used for tumour growth curves. Multiple comparisons *post hoc* tests were chosen based on how many group comparisons were made. When all groups were compared with each other, Tukey's Honestly Significant Difference (HSD) test was used, while Sidak's test was used when individual time points were compared with each other. Non-parametric Mann–Whitney *U*-test/Wilcoxon sign-rank test (for paired comparison) was used to compare two groups, while Kruskal–Wallis test followed by Dunn's multiple comparisons was used for >2 group-wise comparisons. GraphPad Prism 6.0 was used for statistical analyses. The threshold for statistical significance was set at *α*=0.05 (*P<0.05, **P<0.01 and ***P<0.0001).

### Antibodies and growth factors

All antibodies used in this study, their origin and working concentrations are listed in Supplementary Table 1.

Recombinant mouse VEGFA164 was purchased from Peprotech EC Ltd (Rocky Hill, NJ, USA) and used for *in vitro* analyses. Dog VEGFA164, which is 99% identical to mouse VEGFA, was used for *in vivo* analyses and was produced at the Paul-Scherrer Institute, Villigen, Switzerland. *In vitro* tests showed no difference in VEGFR2-stimulating activity of commercial mouse VEGF and in-house-produced dog VEGF. Histamine was from Sigma-Aldrich.

### Orthotopic mouse glioma model

Mice (homozygous *Vegfr2*^*Y949F/Y949F*^ and WT littermates) were inoculated with GL261 glioblastoma cells (a kind gift from Geza Safrany, NRIRR, Budapest). Cells were not verified but tested negative for mycoplasma using the Mycoplasma detection kit (Lonza). GL261 is a chemically induced murine cell line, which when inoculated orthotopically has many features reminiscent to human glioma^18^. Cells were cultured in DMEM Glutamax medium containing 10% FCS, washed and resuspended in PBS for inoculation. Mice were anaesthetized with isoflurane (Isoba, 3% induction and 1.5% maintenance). A small incision was made rostrocaudally along the midline of the scalp. A small hole was drilled at stereotaxic coordinates +1.1 mm anterior and 2.1 mm lateral to bregma. A volume of 0.5 μl of cells (1.5 × 10^5^ cells per μl) were slowly injected using a Hamilton microsyringe with a 33 G needle at 2.9 mm depth below the dura. The needle was left in place for 3 min before slow retraction over 2 min. The wound was closed using 6-0 resorbable suture, and analgesia carprofen (Rimadyl Vet, Orion Pharma, Sweden; 50 μg per 20 g mouse) was injected subcutaneously on the back. Tumours were left to grow for 18 days. On the last day, mice were intravenously injected with 150 μg per 20 g mouse of Alexa-555-Cadaverine (Life Technologies) together with 100 μg of biotinylated tomato lectin in PBS. After 15 min of circulation, mice were killed using an overdose of ketamine/xylazine (see above) and transcardially perfused with physiological saline followed by cold 4% paraformaldehyde (PFA). Brains were postfixed for 4 h and transferred to 25% sucrose for cryoprotection. Brains were coronally cut on a vibratome at 80 μm thickness and mounted on cover glass in series to cover the entire volume of the tumour. Tumour volume was estimated using haematoxylin-and-eosin-stained sections throughout the brain. Representative sections were immunostained for lectin (Streptavidine-Alexa-488) and nuclei (Hoechst). The two largest (mid) sections of each tumour were used for quantification of vessel density and Cadaverine-555 accumulation in the brain, indicating abnormal leakage.

### RipTag spontaneous endocrine tumour and metastasis model

*Vegfr2*^*Y949F/Y949F*^ mice were crossed with *RIP-Tag2* mice on the C57Bl/6 background. *Vegfr2*^*Y949F/Y949F*^ heterozygous offspring was bred to obtain *RIP-Tag2:WT* and *RIP-Tag2:Vegfr2*^*Y949F/Y949F*^ *homozygous* mice. All analyses were done using mice of mixed gender aged 14 weeks. Islets larger than 1 × 1 mm were defined as Riptag tumours, and overtly red islets <1x1 mm were defined as angiogenic islets as assessed under a stereological microscope. Tumours and pancreas including angiogenic islets were sectioned and stained to analyse vessel characteristics. For quantification of metastases, right liver lobes were sectioned in their entirety. In all, 30 consecutive sections were examined for score of metastases based on a cluster of at least 25 cells, which were confirmed by SV40-Tag staining. Average numbers of liver metastases per section was calculated.

### B16F10 subcutaneous primary tumour and metastasis model

B16F10 cells (American Type Culture Collection) were not validated after purchase but retained melanin production and were regularly tested negative for mycoplasma using the Mycoplasma Detection Kit (Lonza). Cells (0.5 × 10^6^ per 100 μl) were injected subcutaneously in the mouse back skin. Tumour size was measured with a caliper every other day from D6 and tumours were collected at D12 or D18 after implantation. Tumour wet and dry weights were measured to determine water content/oedema in the tumours, calculated (tumour wet weight−tumour dry weight)/tumour wet weight and given as percentage (%). Tumour vessel characteristics were analysed after injection of FITC-Lectin (Sigma) in the tail vein, followed by 20-min circulation and then, a washout with PBS via the left cardiac ventricle. Snap-frozen tumours were sectioned, stained and imaged using a Zeiss Axioimager. Quantification of vessel parameters was done using ImageJ (National Institutes of Health) in a blinded fashion. To score for metastatic spread, lentivirus-transduced DsRed-expressing B16F10 cells (kindly provided by Professor David D. Schlaepfer Department of Pathology, La Jolla, CA) were injected in the mouse right ear dermis (4 × 10^4^ cells in 10 μl growth factor-depleted Matrigel; BD). Tumour volume was measured with a caliper every other day. At D15, mice were perfused with PBS via the left cardiac ventricle and lungs were removed, fixed and frozen. For quantification of metastases, the left large lung lobe from each mouse was sectioned in its entirety. Lung metastases were enumerated by counting DsRed-positive lesions from confocal microscopy in four sections spaced 100 μm apart, in each specimen.

### Microsphere extravasation in mouse trachea and Riptag tumours

Twenty microlitres of 30 nm Fluoro-Max Green Aqueous Fluorescent microspheres (Thermo Scientific, Fremont, CA, USA) together with 80 μl solution of VEGFA164 (5 μg per 20 g body weight) or PBS were administered by tail vein injection. Mice were transcardially perfused using 1% PFA in PBS 1 h later. For histamine-induced microsphere extravasation, 20 μl of 30 nm fluorescent microspheres was injected together with histamine solution (80 μl; 1.25 mg per 20 g body weight). Perfusion with 1% PFA was performed after 2 min circulation^45^. Tracheas were dissected and fixed in 1% PFA in PBS for an additional 2 h, stained with endothelial cell markers to visualize the vasculature. To observe microsphere extravasation in Riptag tumours, 100 μl of 30-nm fluorescent microspheres was administered by tail vein injection. Intravascular microspheres were removed by PBS perfusion via the left ventricle 1 h later. Riptag tumours were postfixed in 2% PFA for 6 h and kept in 30% sucrose overnight at 4 °C followed by embedding in cryosectioning medium. Tumours were sectioned and stained for endothelial cell markers. Imaging was performed using a Zeiss700 confocal microscope or a Zeiss 710 Structured Illumination microscope.

### TMZ chemotherapy

B16F10 cells (0.5 × 10^6^ per 100 μl) were injected subcutaneously in the mouse back skin. From D4 to D8, TMZ dissolved in dimethylsulfoxide to a final concentration of 2%, was administered i.p. every day at 10 mg kg^−1^, a dose established to have no effect on B16F10 growth in WT mice from test studies. Control mice were treated with a similar volume of 2% dimethylsulfoxide. Tumour size was measured with a caliper every other day from D6 until D18 after implantation.

### DsRed-expressing B16F10 cell lung colonization

DsRed-expressing B16F10 cells (1 million in 100 μl PBS) were injected into mouse tail vein. At 48 h, mice were perfused with PBS via the left cardiac ventricle, and lungs were removed, fixed and frozen. Left lung lobes were sectioned in their entirety. Tumour cell foci were enumerated by counting DsRed-positive lesions in confocal microscopy in two sections spaced 400 μm apart and five fields per section.

### Miles assay

Mice (females, 6–8 weeks) were subjected to i.p. injection with pyrilamine maleate salt (4 mg per kg body weight in 0.9% saline, Sigma) to inhibit unspecific histamine release (for example, by pulling the skin), 30 min before Evans' blue tail vein injection (100 μl 1% Evans blue in sterile saline, Sigma). This was followed by intradermal injection of VEGF (100 ng in 50 μl per site) or sterile saline 10 min after Evans' blue administration. Thirty minutes after the VEGF injection, the dorsal skin was excised and Evans' blue was extracted by immersion in formamide buffer. Absorbance at 620 nm was measured using a spectrophotometer (EMax microplate reader, Molecular Devices, CA, US). Extravasated Evans' blue was determined from a standard curve and normalized to tissue weight (g).

### Isolation of lung endothelial cells

Mouse lungs were collected at P10 or 3–4 weeks, minced and digested in 10 ml Dulbecco's PBS medium containing 2 mg ml^−1^ collagenase type I (Sigma), for 1 h at 37 °C with shaking, followed by filtration through a 70-μm disposable cell strainer (BD Falcon). Cells were centrifuged at 400*g* for 8 min at 4 °C, suspended in cold PBS with 0.1% bovine serum albumin (BSA) and incubated with anti-rat immunoglobulin G-coated magnetic beads (Dynabeads sheep anti-Rat IgG, Invitrogen) pre-coupled with rat anti-mouse platelet/endothelial cell adhesion molecule-1 (PECAM-1; MEC13.3, BD Pharmingen, 553370) for 10 min, with gentle agitation. Beads were separated from the solution with a magnetic particle concentrator (Dynal MPC-S, Invitrogen). The beads were washed five times with PBS, and endothelial cells were suspended in MV2 growth medium with the following supplements: 5 ng ml^−1^ endothelial growth factor; 0.2 μg ml^−1^ hydrocortisone; 0.5 ng ml^−1^ VEGFA; 10 ng ml^−1^ basic fibroblast growth factor; 20 ng ml^−1^ insulin-like growth factor-1 (Promocell); and 5% penicillin/streptomycin (Sigma). The purity of cells was >95% as determined by staining for VE-cadherin. The isolated lung endothelial cells were cultured on slides pre-coated with gelatin. Cells were treated with or without 100 ng ml^−1^ mouse VEGFA164 for 10 min before fixation in 4% PFA in PBS for 10 min, permeabilized in 0.1% Triton X-100 in PBS for 30 min, followed by immunofluorescent staining. Images were acquired using a Zeiss LSM700 confocal microscope (× 63, numerical aperture 1.4 objectives).

### Immunoprecipitation and immunoblotting

For *in vitro* signal transduction analyses, endothelial cells were isolated from lungs of P10 pups as described above, and cultured. Cells were treated with or without 100 ng ml^−1^ mouse VEGFA164 (PeproTech) for 8 min and lysed for subsequent immunoprecipitation and immunoblotting.

For *in vivo* signal transduction analyses, lung lysates were used from mice at 1, 2, 5, 10, 15 or 60 min after injection of dog VEGFA164 in the tail vein. An equal volume of PBS was injected into control mice. B16F10 tumour lysates were obtained from mice at D12 or 18 after B16F10 cell implantation. Tissues were lysed in commercial RIPA buffer (ProteinSimple) followed by immunoprecipitation for VEGFR2 and immunoblotting, or immunoblotting of total lysates. For immunoprecipitation, 1.5 mg/ml tissue lysate were diluted in RIPA buffer (20 mM HEPES, pH 7.5, 150 mM NaCl, 1% NP 40) supplemented with protease inhibitors (Roche, 04693116001), phosphatase inhibitors (Thermo scientific, 88667), 1 mM NaF, 1 mM Na3VO4. Lysates were precleared by incubating with 25 μl of Protein G Sepharose 4 Fast Flow beads (Ge Healthcare, 17-0618-01) per sample for 2 h at 4 °C. Precleared lysates were incubated with 1.5 μg VEGFR2 antibody for 2 h at 4 °C and 25 μl of Protein G beads were added and incubation continued for 1 h (lung lysates) and overnight (B16 tumor lysate and isolated lung endothelial cells). Sepharose beads were washed 5 times with 1 ml RIPA buffer per sample without protease and phosphatase inhibitors and collected by centrifugation at 3000Xg for 1 min at 4 °C. 30 μl of 2X LDS sample buffer were added to each sample and heat at 70 °C for 10 min. Immunocomplexes were resolved on NuPAGE Novex 4–12% Bis-Tris gels (Invitrogen) and processed for immunoblotting^8^. Full scans of all immunoblots are presented in Supplementary Figs 6–13.

### Immunofluorescent staining

Eyes were removed and prefixed in 4% PFA for 20 min at room temperature. After dissection, retinas were blocked overnight at 4 °C in 1% FBS (Gibco), 3% BSA (Sigma) and 0.5% Triton X-100 (Sigma). Retina samples were incubated over night with primary antibodies in blocking reagent, followed by washing and incubation with Isolectin-B4 in Pblec (1 mM MgCl_2_, 1 mM CaCl_2_, 0.1 mM MnCl_2_ and 1% Triton X-100 in PBS) overnight. Thereafter, samples were incubated with the appropriate secondary antibody for 2 h at room temperature, and mounted in fluorescent mounting medium (DAKO). Images were acquired using a Zeiss700 confocal microscope. For analysis of the activation state of endothelial junctions in the retinal vasculature, the unbiased software (Matlab)^15^ was used.

### Blood parameter measurements

The blood flow measurements were performed with a microsphere technique^46^ as described below: WT and *Vegfr2*^*Y949F/Y949F*^ mice 3–4 months old were anaesthetized with isoflurane and placed on an operating table, where the animal's body temperature was maintained constant at 38 °C. A polyethylene tracheal tube was inserted to ensure free airways and polyethylene catheters were inserted into the ascending aorta, via the right carotid artery, and into the right femoral artery. The former catheter was connected to a pressure transducer (Physiologic Pressure Transducer; AD Instruments, Oxford, UK) to allow continuous monitoring of the mean arterial blood pressure, which was allowed to stabilize for 20–25 min. Approximately 10^5^ microspheres (EZ-Trac; Triton Technology, San Diego, CA, USA) with a diameter of 10 μm were injected for 10 s via the catheter with its tip in the ascending aorta. Starting 5 s before the microsphere injection, and continuing for a total of 60 s, an arterial blood reference sample was collected by free flow from the catheter in the femoral artery at a rate of 0.10 ml min^−1^. The exact withdrawal rate in each experiment was confirmed by weighing the sample.

Arterial blood was then collected from the catheter in the femoral artery and analysed for blood pH, pCO_2_, pO_2_, oxygen saturation, S-HCO_3_^−^, base excess, S-Na^+^, S-K^+^, haematocrit and haemoglobin concentration, with an iStat (Abbott Scandinavia). The cassette used for this analysis requires 90 μl of whole blood. Arterial blood was also used for the determination of blood glucose concentrations (Medisense Test Reagent Strips; MediSense) and for serum insulin determinations with ELISA (Rat Insulin ELISA; Mercoedia AB).

The animals were killed, and the organs were carefully dissected free from fat, blotted and weighed. The microspheres in the organs and the arterial blood reference sample were visualized by a freeze-thawing technique and counted by an observer blinded for the origin of the samples^47^.

The blood flow values were calculated according to the formula *Q*_org_=*Q*_ref_ × *N*_org_/*N*_ref_, where *Q*_org_ is organ blood flow (ml min^−1^), *Q*_ref_ is withdrawal rate of the reference sample (ml min^−1^), *N*_org_ is the number of microspheres present in the organ and *N*_ref_ is the number of microspheres present in the reference sample^46^.

### Blood pressure measurements after VEGF administration

Mice (females, ∼15 weeks) were anaesthetized by an intraperitoneal (i.p.) injection of avertin^48^. A polyethylene tracheal tube was inserted to ensure free airways, and a polyethylene catheter was inserted into the descending aorta, via the right femoral artery, and connected to a pressure transducer (AD Instruments) connected to a PowerLab system (AD Instruments. When the blood pressure had remained stable for 10 min an injection of 0.1 ml of saline or VEGFA (5 μg per 20 g body weight) was given in the right jugular vein. Mean arterial blood pressure was then registered for 15 min.

### E11.5 Hindbrain vascularization

Embryos at E11.5 were collected and fixed in 4% PFA overnight. Hindbrains were dissected the following day and thereafter, brains were permeabilized in a buffer containing 1% Triton X, 0.01 mM CaCl_2_, 0.1 mM MgCl_2_, 0.1 mM MnCl_2_, in PBS (pH 6.8), for two 15-min washes and subsequently incubated with biotinylated isolectin-B4 (L-2140, Sigma-Aldrich, Sweden). A streptavidine-coupled Alexa-488 (Life Technologies) was used for visualization. Hindbrains were flat-mounted and imaged in a confocal (LSM700). The image analysis was done using CellProfiler (www.cellprofiler.org) with a pipeline as follows: an initial segmentation of the vessels was done using a fixed global threshold, followed by filtering out small particles. Next, a skeleton was computed from the binary vessel mask. From the skeleton, the number of branch points and the branch lengths were measured. Branches touching the border or very short branches were removed. For each branch, the mean distance values for the corresponding skeleton segment were calculated.

### Transmission electron microscopy

Two sex- and age-matched mice (7-week-old male and female *Vegfr2*^*Y949F/Y949F*^ and WT littermates) were perfusion fixed and their organs were collected and embedded in epoxy resin^16^. Briefly, mice were anaesthetized using a mixture of ketamine/xylazine and perfused via the left heart ventricle with oxygenated DMEM/1 mM CaCl_2_/5 mM D-glucose. This was immediately followed by perfusion with 3% PFA/1.5% glutaraldehyde/5% sucrose in 0.1 M phosphate buffer, pH 7.2. Organs (kidney and pancreas) were removed, cut in small blocks and further fixed (1 h at room temperature) in 2% glutaraldehyde in 0.1 M sodium cacodylate buffer, rinsed in 0.1 M cacodylate, postfixed (1 h, on ice, in the dark) in 1% OsO4, stained (over night, room temperature, in the dark) with 2% uranyl acetate, dehydrated in graded ethanol and LX-112 embedded (Ladd Research, Burlington, VT). Ultrathin sections (∼30 nm) were obtained using an oscillating diamond knife (Diatome, Emsdiasum), further stained with 2% uranyl acetate followed by lead citrate, examined and imaged with a Jeol 1010 electron microscope using a bottom-mount AMD camera. Images were exported in TIFF format, and labelled and assembled using Adobe Photoshop and Illustrator CS6 (Adobe Systems). In some images, the contrast and brightness were post-processed in Adobe Photoshop using the respective linear functions.

### RNA extraction and sequencing

RNA was extracted using the Qiagen RNeasy kit following the manufacturer's instructions and using lungs from 5- to 8-week-old mice. Extracted RNA quality was assessed with a BioAnalyzer (Agilent Technologies, Wilmington, DE, USA) and concentration estimated using the Qubit RNA BR Assay Kit (Thermo Ficher Scientific, Eugene, Oregon, USA). From 11 μg of total RNA, mRNA purification was performed using Dynabeads mRNA Purification Kit (Ambion, Life Technologies AS, Oslo). mRNA libraries, three biological replicates per sample, were prepared using the Ion Total RNA-Seq Kit v2 (Life Technologies) using the standard protocol, and sequencing was performed on Ion Proton sequencer (Life Technologies). Libraries were barcoded three per PI v2 chip to achieve 20 million reads per replicate. Reads were processed, mapped to a reference genome (mm10) and summarized using Torrent Suite version 5.0.2 and default settings. The statistical analysis of gene expression was performed in R using DESeq2 package (doi:10.1186/gb-2010-11-10-r106) with dispersion fit type set to local. The FPKM (fragments per kilobase per million) function from DESeq2 package was used to normalize reads per gene as RPKM (reads per kilobase per million), using the robust library size estimate. The RPKM values for selected genes were plotted on the log10 scale based on the folds of our genes of interest established in log2 fold change (log2FC=1 means the gene is upregulated twofolds).

## Additional information

**How to cite this article:** Li, X. *et al*. VEGFR2 pY949 signalling regulates adherens junction integrity and metastatic spread. *Nat. Commun.* 7:11017 doi: 10.1038/ncomms11017 (2016).

## Supplementary Material

Supplementary Information Supplementary Figures 1-13, Supplementary Table 1

## Figures and Tables

**Table 1:** Haemodynamics in WT and *Vegfr2* ^*Y949F/Y949F*^ mice.

Strain	WT (*n*=9)	Y949F (*n*=8)	*P* value
pH	7.29±0.01	7.32±0.02	0.288
Arterial pCO_2_ (kPa)	5.8±0.3	5.5±0.3	0.596
Arterial pO_2_ (kPa)	13.1±0.9	14.7±0.6	0.167
Base excess (mmol l^−1^)	−5.8±0.6	−4.6±1.0	0.341
Arterial HCO_3_ (mmol l^−1^)	20.7±0.7	21.3±1.1	0.661
Arterial oxygen saturation (%)	95.6±1.0	97.5±0.4	0.185
S-Na^+^ (mmol l^−1^)	144.8±0.6	143.2±0.5	0.09
S-K^+^ (mmol l^−1^)	5.1±0.2	5.2±0.2	0.757
Haematocrit (%)	32.3±1.1	33.0±1.6	0.73
Haemoglobin (g l^−1^)	110±4	112±5	0.732
			
Blood flow values*			
Pancreatic			
Whole gland	1.76±0.36	1.42±0.22	0.458
Caput	2.10±0.48	1.47±0.20	0.532
Cauda	1.52±0.32	1.38±0.24	0.735
Duodenal	4.57±1.21	3.60±0.70	0.514
Colonic	1.51±0.24	1.48±0.47	0.361
Lung	0.15±0.07	0.21±0.09	0.665
Kidney	7.15±1.65	5.69±1.28	0.503
CNS	1.07±0.18	1.64±0.48	0.47
Fat	0.013±0.004	0.008±0.003	0.768

CNS, central nervous system.

Values are means±s.e.m.

^*^ml min^−1^ × g organ weight.
