# Supplementary material for: VEGFR2 pY949 signalling regulates adherens junction integrity and metastatic spread
Source: Nat Commun. 2016 Mar 23;7:11017. doi: 10.1038/ncomms11017 (PMC4814575; doi:10.1038/ncomms11017)
Supplement: Supplementary Information — Supplementary Figures 1-13, Supplementary Table 1 [file ncomms11017-s1.pdf]

Supplementary Information

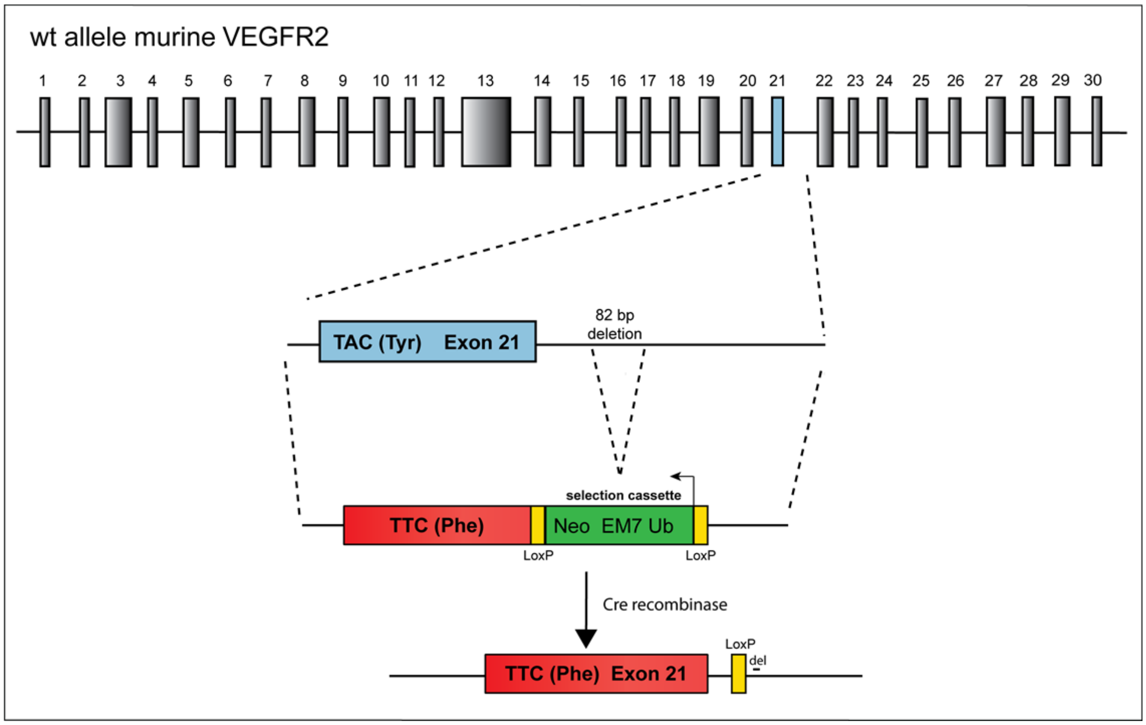

Supplementary Fig. 1. Generation of *Vegfr2*$^{Y949F/Y949F}$ mice.

Velocigene technology was used to generate the exchange of amino acid Y949 (TAC) for Phe (TTC) in the 5´sequence corresponding to exon 21 flanking a LoxP-restricted Neomycin cassette. The sequence exchange was introduced through homologous recombination into F1H4 embryonic stem cells of background 129S6SvEv/C57Bl6F1. The cassette was removed by treatment with Cre recombinase, resulting in an 82 bp deletion in intron 21. The correct introduction of the mutation and the otherwise unaffected sequence of exon 21 and 22 was verified by nucleotide sequencing on both strands on DNA from tail biopsy from *Vegfr2*$^{Y949F/Y949F}$ mice. Mice were back-crossed onto C57Bl6 background for more than 10 generations before being used for analyses described in the accompanying study by Li, Padhan et al.

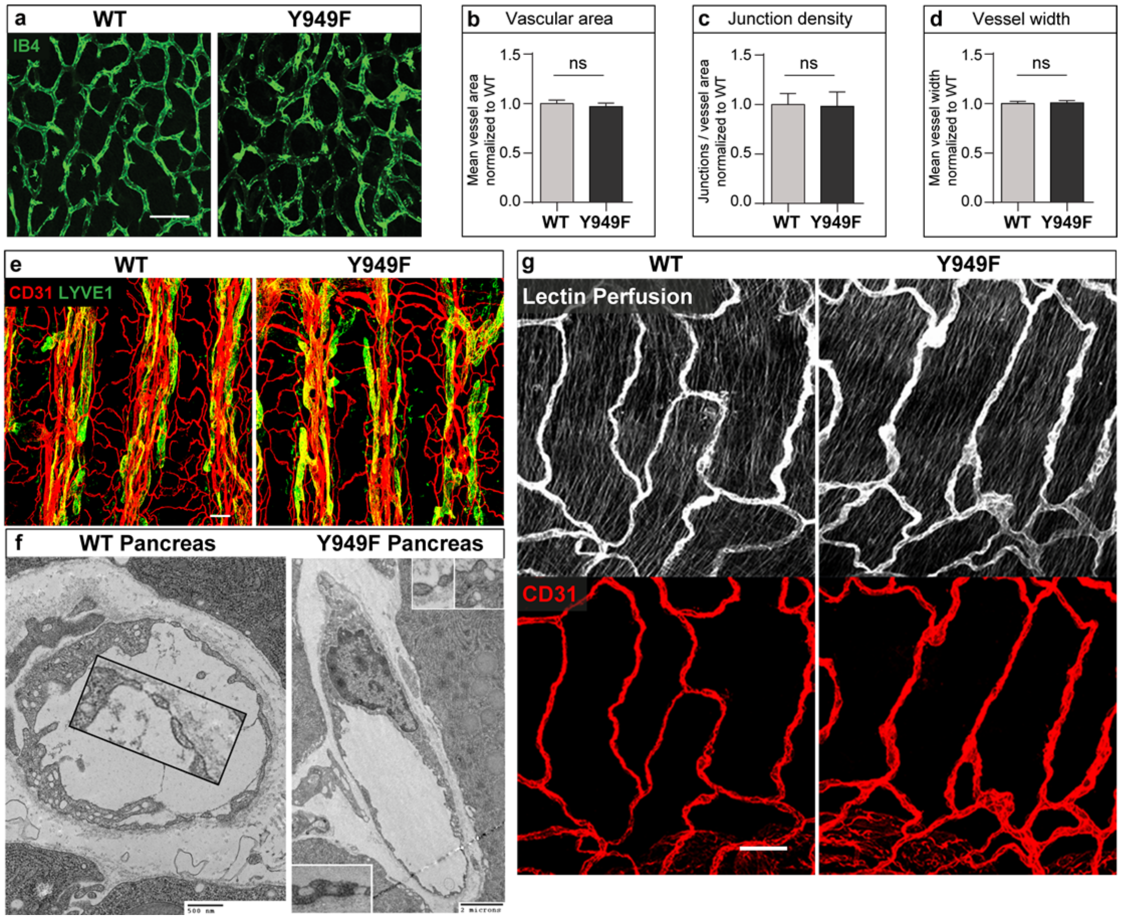

Supplementary Fig. 2. <u>Unaffected development and flow in the *Vegfr2*$^{Y949F/Y949F}$ mouse.</u>

a. WT and *Vegfr2*$^{Y949F/Y949F}$ (Y949F) embryos harvested at embryonic day (E) 11.5 were subjected to immunostaining for isolectin B4.

b-d. Quantification for junction density (b), vascular area (c) and vessel width (d). Y949F data were normalized to WT. n=6-7 mice/genotype from 5 matched litters. Student's test, ns; not significant. Data in b-d are presented as mean ± SEM. Scale bar, 50 μm.

e. Tracheal lymphatics visualized by immunostaining for CD31 (red) and LYVE1 (green). n=3; performed once. Scale bar, 50 μm.

f. Transmission electron microscopy (TEM) ultrastructural analysis of WT and Y949F pancreas showing fenestrated vessels (insets) with similar morphology in the two genotypes. Similar results were obtained in 3 independent analyses. Scale bars, 500 nm (left) and 2 μm (right).

g. Vessel perfusion in WT and *Vegfr2*$^{Y949F/Y949F}$ (Y949F) mice. Mice were tail vein- injected with FITC-lectin and tracheas harvested after 20 min circulation followed by CD31 immunostaining. The lectin distributed similarly in the two genotypes. n= 2; performed twice. Scale bar; 50 μm.

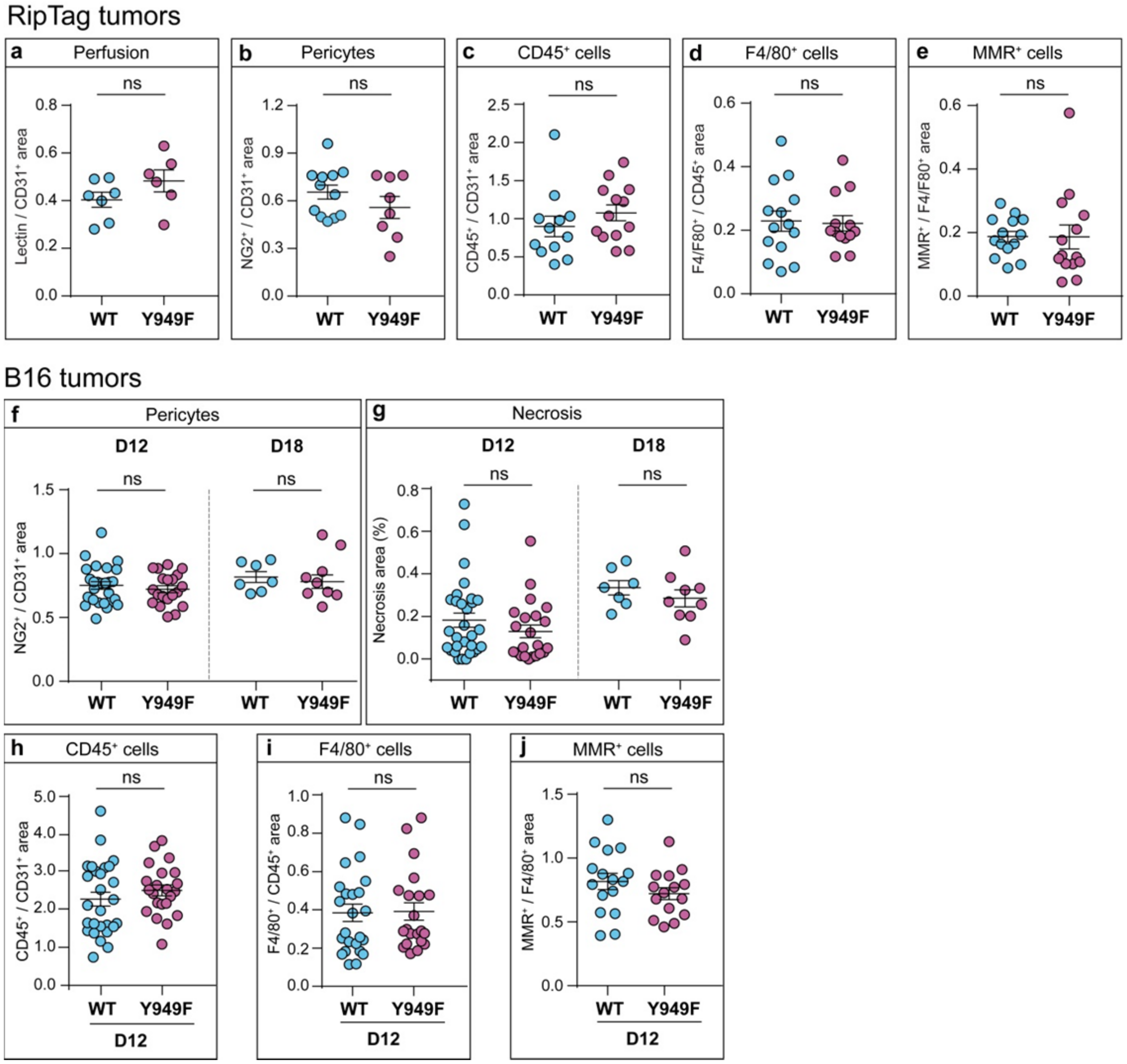

Supplementary Fig. 3. Tumor microenvironment parameters in RipTag insulinomas and B16F10 melanomas.

a. Perfusion in RipTag insulinomas was estimated by lectin perfusion followed by immunostaining for CD31. There was no difference in the extent of perfusion in insulinomas from WT and *Vegfr2*$^{Y949F/Y949F}$ RipTag mice. n=6-7 mice/genotype. Student's t-test, ns; not significant, performed once.

b. Pericytes surrounding insulinoma vessels in WT and *Vegfr2*$^{Y949F/Y949F}$ RipTag tumors was determined by immunostaining for nerve-glia2 (NG2). The NG2-positive area was normalized to CD31 area. There was no difference between the genotypes. n=8-12 mice/genotype, multiple tumors/mouse. Student's t-test, ns; not significant, performed once.

c-e.Inflammatory cells infiltrating insulinomas in WT and *Vegfr2*$^{Y949F/Y949F}$ RipTag mice. The areas of tumor-infiltrating CD45+ (c) F4/80+ (d) or Macrophage mannose receptor (MMR)+ (e) did not differ between the genotypes. n=12-13 tumors/genotype in c; n=13-14

tumors/genotype in d, and n=14 tumors/genotype in e. Student's t-test, ns; not significant, performed once, multiple tumors/mouse.

f. B16F10 tumors from WT and *Vegfr2*$^{Y949F/Y949F}$ mice harvested at day 12 (D12) or D18 after inoculation, analyzed by NG2 immunostaining to determine the extent of pericyte-association with the tumor vasculature. NG2-positive area was assessed in relation to CD31-positive area, and did not differ between the genotypes. n=20-26 mice/genotype at D12 and n=7-8 mice/genotype at D18. Student's t-test, ns; not significant, performed 3 times for D12 and 1 time for D18.

g. Necrosis area (% of field area) was estimated in hematoxylin/eosin-stained sections. The extent of tumor necrosis did not differ between WT and *Vegfr2*$^{Y949F/Y949F}$ at D12 but increased in both conditions with time. n=22-30 mice/genotype at D12 and n=7-8 mice/genotype at D18. Student's t-test, ns; not significant, performed 3 times for D12 and 1 time for D18.

h-j. Quantification of tumor-infiltrating CD45+ (h) F4/80+ (i) or MMR+ (j) cells in tumors did not differ between the genotypes. n=22-27 mice/genotype (h), 24-27/genotype (i) and 15-16 mice/genotype (j). Student's t-test, ns; not significant, performed 3 independent times for (h-i) and 2 times for (j).

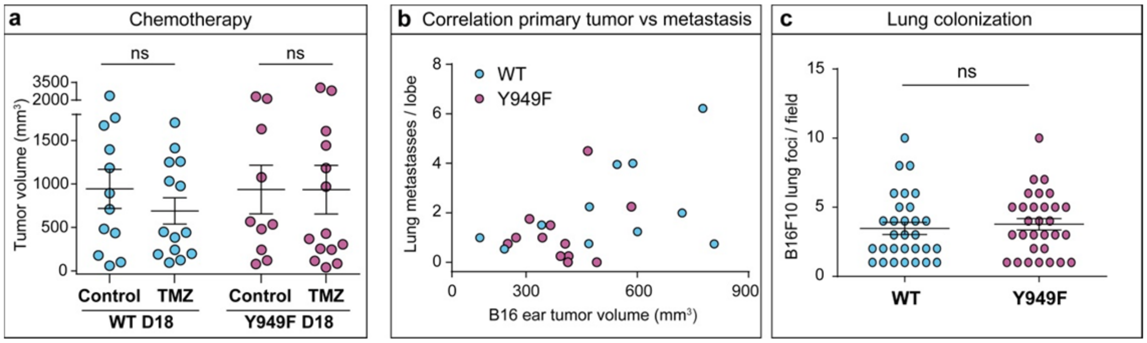

Supplementary Fig. 4. Effect of Y949F mutation on B16F10 properties.

a. Effect of Temozolomide (TMZ) on volumes of primary B16F10 tumors at day 18 (D18). Mice were treated with TMZ or vehicle (DMSO) between D4 and D8 after inoculation. Student's test, ns; not significant. Data are presented as mean ± SEM.

b. Correlation analysis shows no relationship between primary B16F10 tumor growth and spontaneous lung metastasis; analysis of data shown in main Fig. 5h and i. n=12-13 mice/genotype. Pearson's correlation; WT, r^2=0.22, *Vegfr2*$^{Y949F/Y949F}$, r^2=0.06. Based on one representative experiment.

c. Similar extent of colonization of lungs by dsRedB16F10 tumor cells injected into the tail vein of WT and *Vegfr2*$^{Y949F/Y949F}$ (Y949F) mice; 48 h after injection. n=30 sections each from 3 mice/genotype. ns; not significant. Data are presented as mean ± SEM; Student's t-test. Based on one representative experiment.

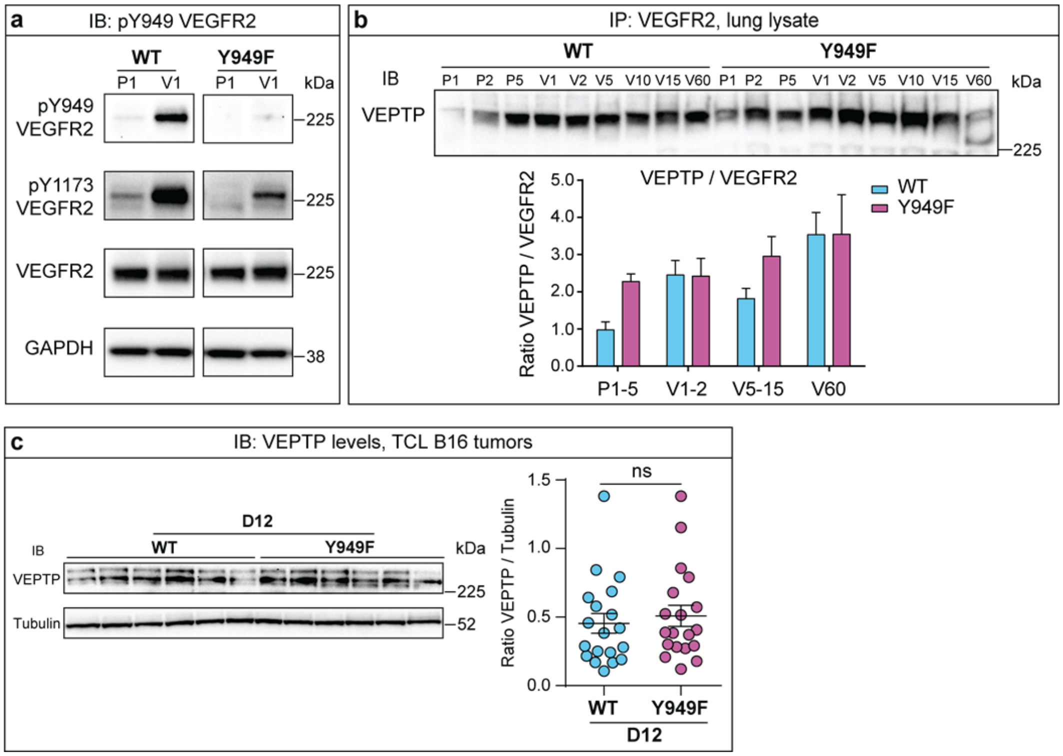

Supplementary Fig. 5. VEGFR2 signaling in WT and in *Vegfr2*$^{Y949F/Y949F}$ mice.

a. VEGFA-induced activation of VEGFR2 and phosphorylation of Y949 and Y1173. WT and *Vegfr2*$^{Y949F/Y949F}$ (Y949F) mice were injected with VEGFA in the tail vein and lungs were harvested after 1 min circulation, followed by lysis and immunoblotting for VEGFR2 phosphotyrosines Y949 and Y1173. Immunoblotting was performed for VEGFR2 and GAPDH to control for equal loading. Representative blot is shown. Note that Y949 was phosphorylated only in the WT. Background in the Y949F lysate may be due to crossreactivity of the anti-pY949 antibody with unphosphorylated receptor. Performed 3 independent times.

b. VEGFA-induced complex formation between VEGFR2 and VEPTP. VEGFR2 was immunoprecipitated from WT and *Vegfr2*$^{Y949F/Y949F}$ mouse lungs harvested from mice injected with PBS (P) followed by circulation for 1, 2 or 5 minutes, or VEGFA (V) followed by different circulation periods (1, 2, 5, 10, 15 and 60 min). Lung lysates were used for immunoprecipitation of VEGFR2 followed by immunoblotting for VEGFR2 (as shown in main Fig. 6a) or VEPTP. The quantification shows VEGFR2/VEPTP complexes normalized to mean values in the PBS samples from all 4 independent repeats (12 samples). Two-way ANOVA p(genotype)=0.0658, p(time)=0.0037.

c. VEPTP expression levels in B16F10 tumors. VEPTP expression was estimated by immunoblotting on B16F10 tumor lysates harvested at D12 from WT and *Vegfr2*$^{Y949F/Y949F}$ (Y949F) mice; band intensities were normalized to tubulin, analyzed in parallel. There was no change in VEPTP expression levels with tumor progression. n=19, pooled from 3 independent studies. Student's t-test, ns; not significant.

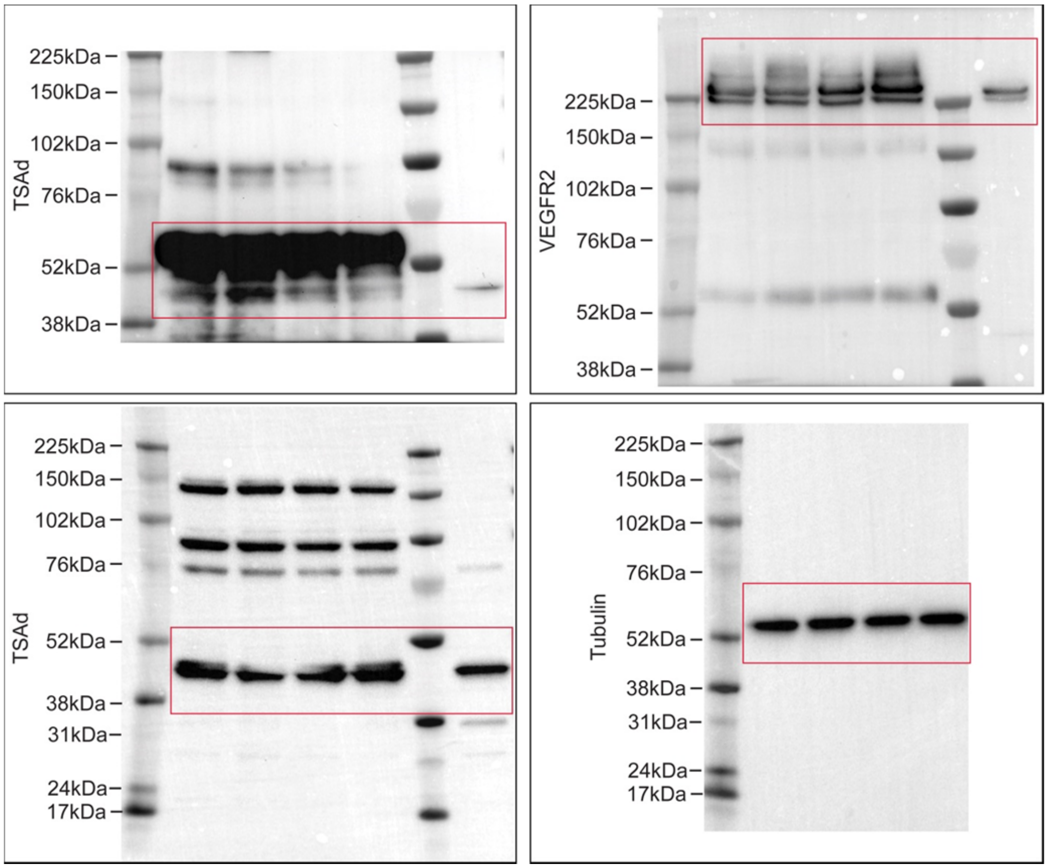

Supplementary Fig. 6. <u>Uncropped immunoblots complementing blot shown in Fig. 1f.</u>

Panels used in Fig. 1f are marked with a red square. For this and the following supplementary figures showing uncropped blots. Molecular weight markers were detected colorimetrically using the ChemiDoc™ MP Imaging System (Bio-Rad); their migration positions are indicated to the left.

To generate samples, isolated endothelial cells treated with VEGFA or not, were subjected to VEGFR2 immunoprecipitation and blotting for TSAd (upper left) or VEGFR2 (upper right). Alternatively, total cell lysate was separated and blotted for TSAd (lower left) or tubulin (lower right). Molecular weight markers are indicated to the left. See Fig. 1f in Li, Padhan et al. for details.

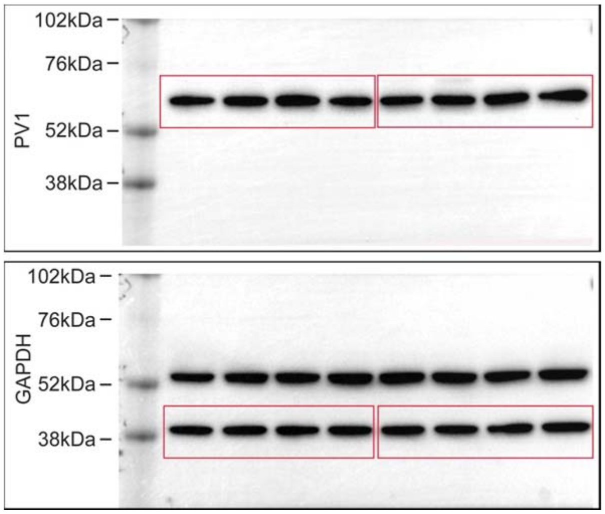

Supplementary Fig. 7. Uncropped immunoblots complementing blot shown in Fig. 2i.

Panels used in Fig. 2i are marked with a red square. Lung lysates were prepared after tail vein injection of PBS or VEGFA and circulation for 1-5 min, followed by immunoblotting for PV1 (upper) and GAPDH (lower). Molecular weight markers are indicated to the left. See Fig. 2i in Li, Padhan et al. for details.

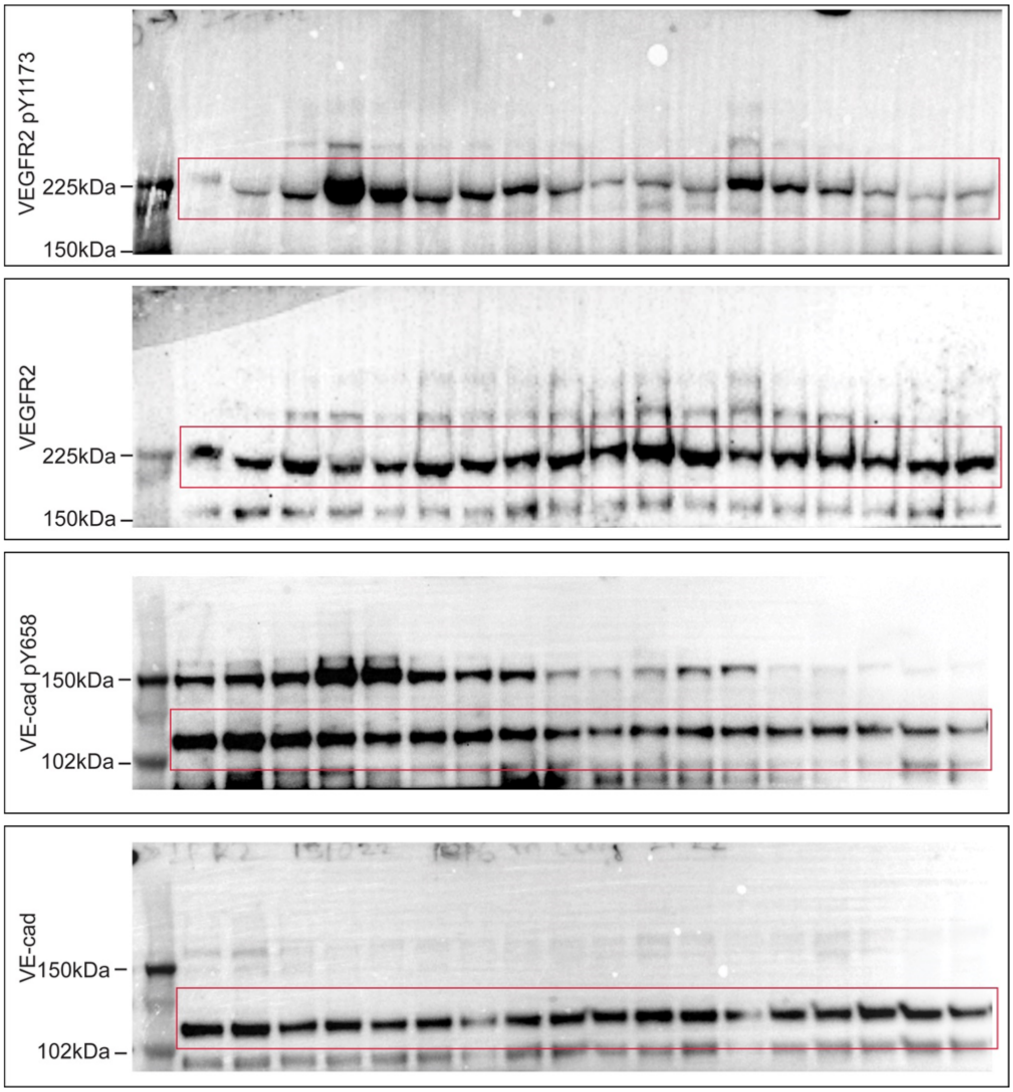

Supplementary Fig. 8. Uncropped immunoblots complementing blots shown in Fig. 6a.

Panels used in Fig. 6a are marked with a red square. Mice (WT or *Vegfr2*$^{Y949F/Y949F}$) were tail vein-injected with PBS or VEGFA followed by circulation for different time periods and preparation of lung lysates that were used for immunoprecipitation of VEGFR2 and immunoblotting for VEGFR2pY1173 (uppermost), VEGFR2 (middle upper), VE-cadherin pY658 (middle lower) and VE-cadherin (lower). Molecular weight markers are indicated to the left. See Fig. 6a in Li, Padhan et al. for details.

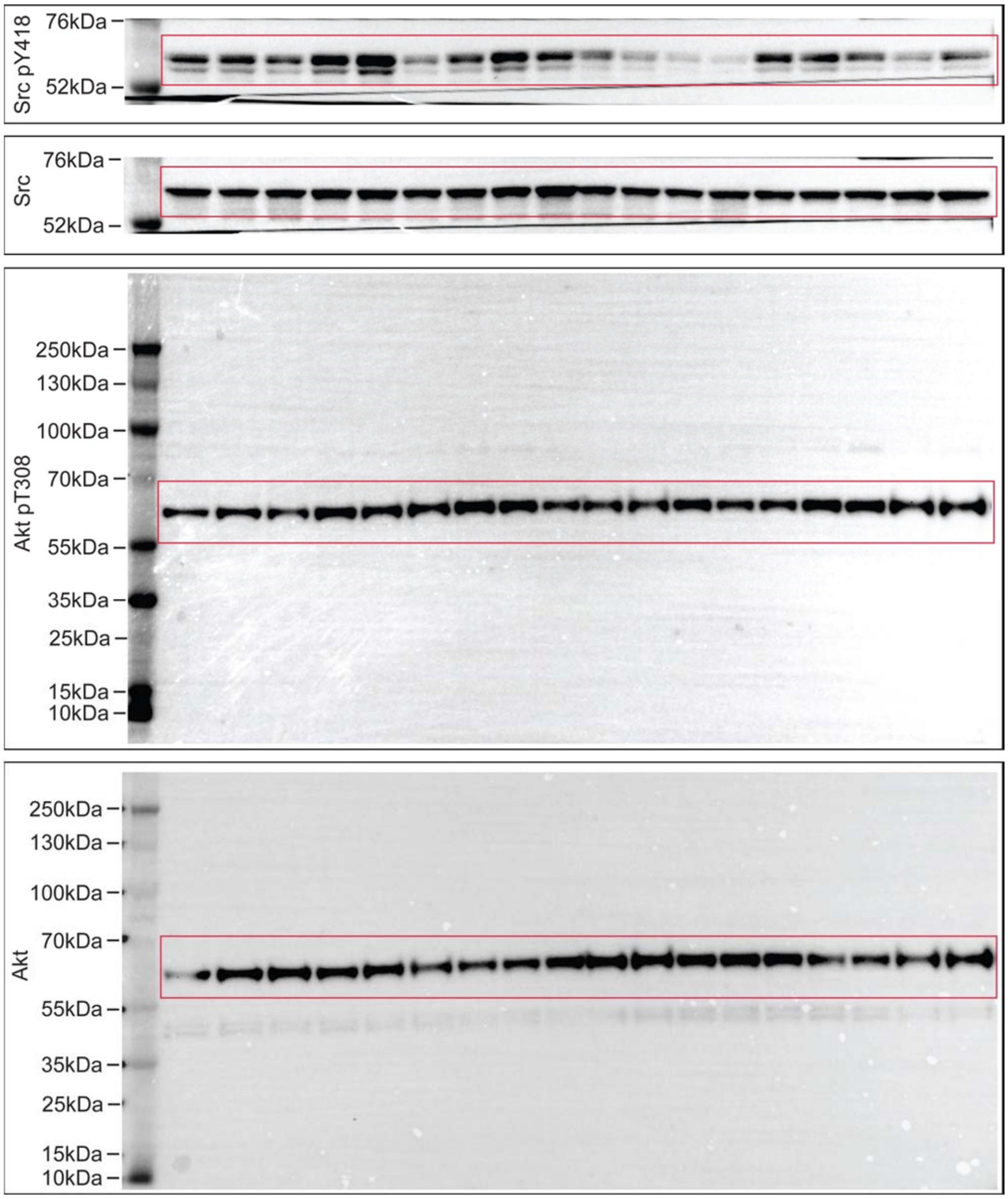

Supplementary Fig. 9. Uncropped immunoblots complementing blots shown in Fig. 6b.

Panels used in Fig. 6b are marked with a red square. Mice (WT or *Vegfr2*$^{Y949F/Y949F}$) were tail vein-injected with PBS or VEGFA followed by circulation for different time periods and preparation of total lung lysates that were used for immunoblotting for c-Src pY418 (uppermost), c-Src (middle upper), Akt pT308 (middle lower) and Akt (lower). Molecular weight markers are indicated to the left. See Fig. 6b in Li, Padhan et al. for details.

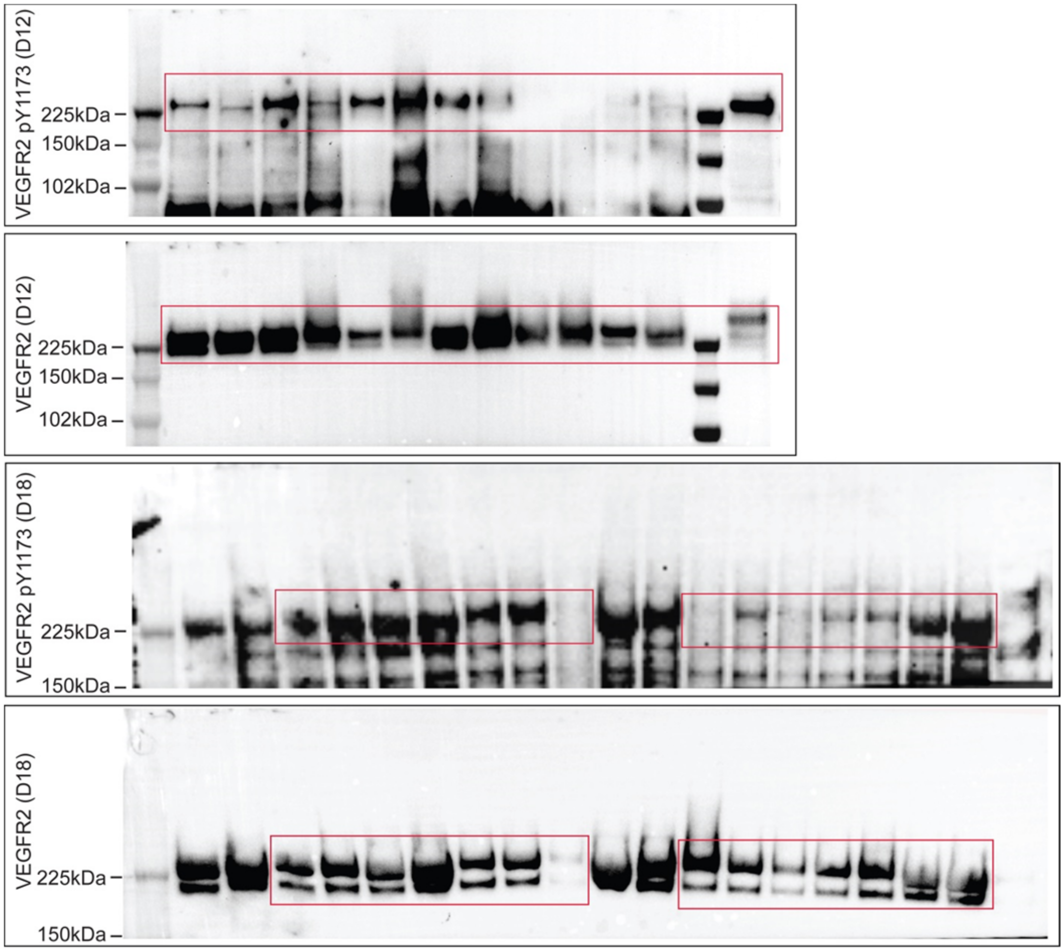

Supplementary Fig. 10. Uncropped immunoblots complementing blots shown in Fig. 6c.

Panels used in Fig. 6c are marked with a red square. B16F10 melanomas from WT or *Vegfr2*$^{Y949F/Y949F}$ mice were harvested at day 12 (D12) or D18 after inoculation, lysed and used for immunoprecipitation of VEGFR2 followed by immunoblotting for VEGFR2 pY1173 (D12, uppermost), VEGFR2 (D12, upper middle), VEGFR2 pY1173 (D18, lower middle) and VEGFR2 (D18, lowermost). Molecular weight markers are indicated to the left. See Fig. 6c in Li, Padhan et al. for details.

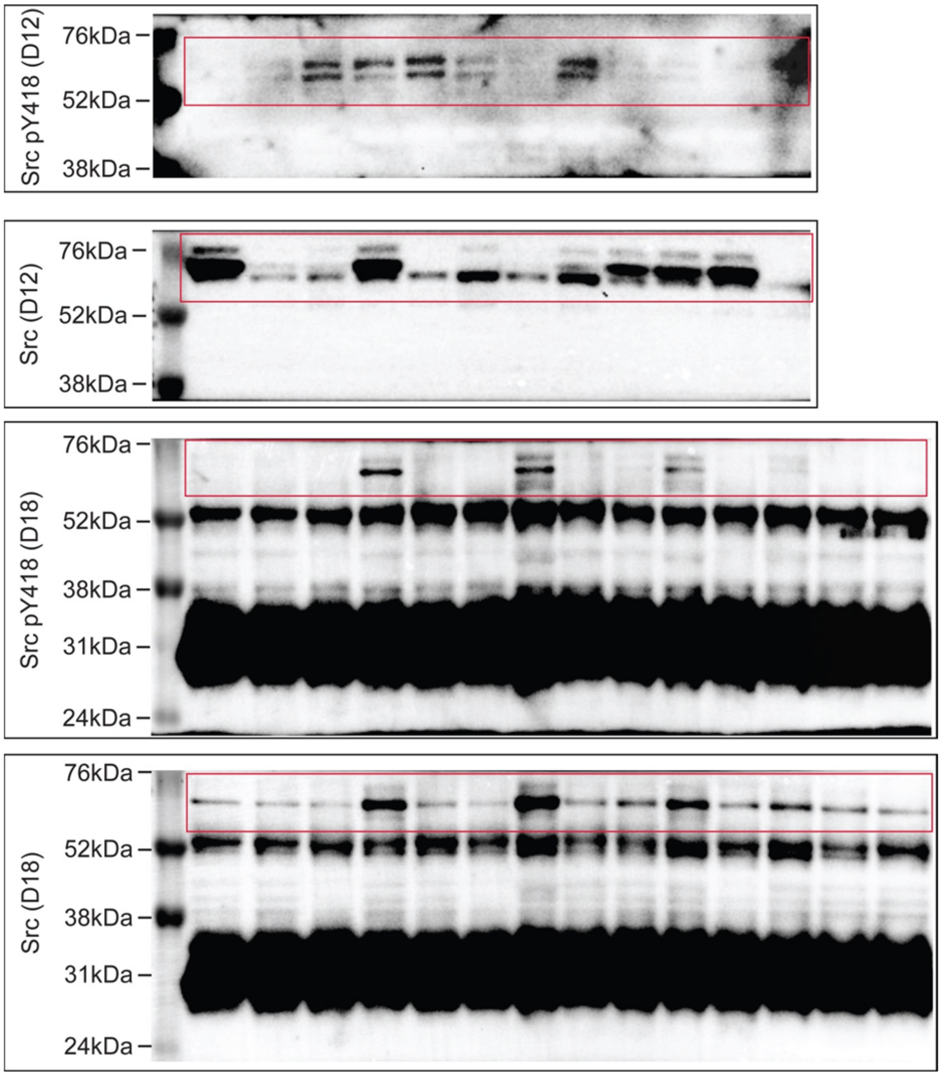

Supplementary Fig. 11. Uncropped immunoblots complementing blots shown in Fig. 6d.

Panels used in Fig. 6d are marked with a red square. B16F10 melanomas from WT or *Vegfr2*$^{Y949F/Y949F}$ mice were harvested at day 12 (D12) or D18 after inoculation, lysed and used for immunoprecipitation of VEGFR2 followed by immunoblotting for Src pY418 (D12, uppermost), c-Src (D12, upper middle), Src pY418 (D18, lower middle) and c-Src (D18, lowermost). Molecular weight markers are indicated to the left. See Fig. 6d in Li, Padhan et al. for details.

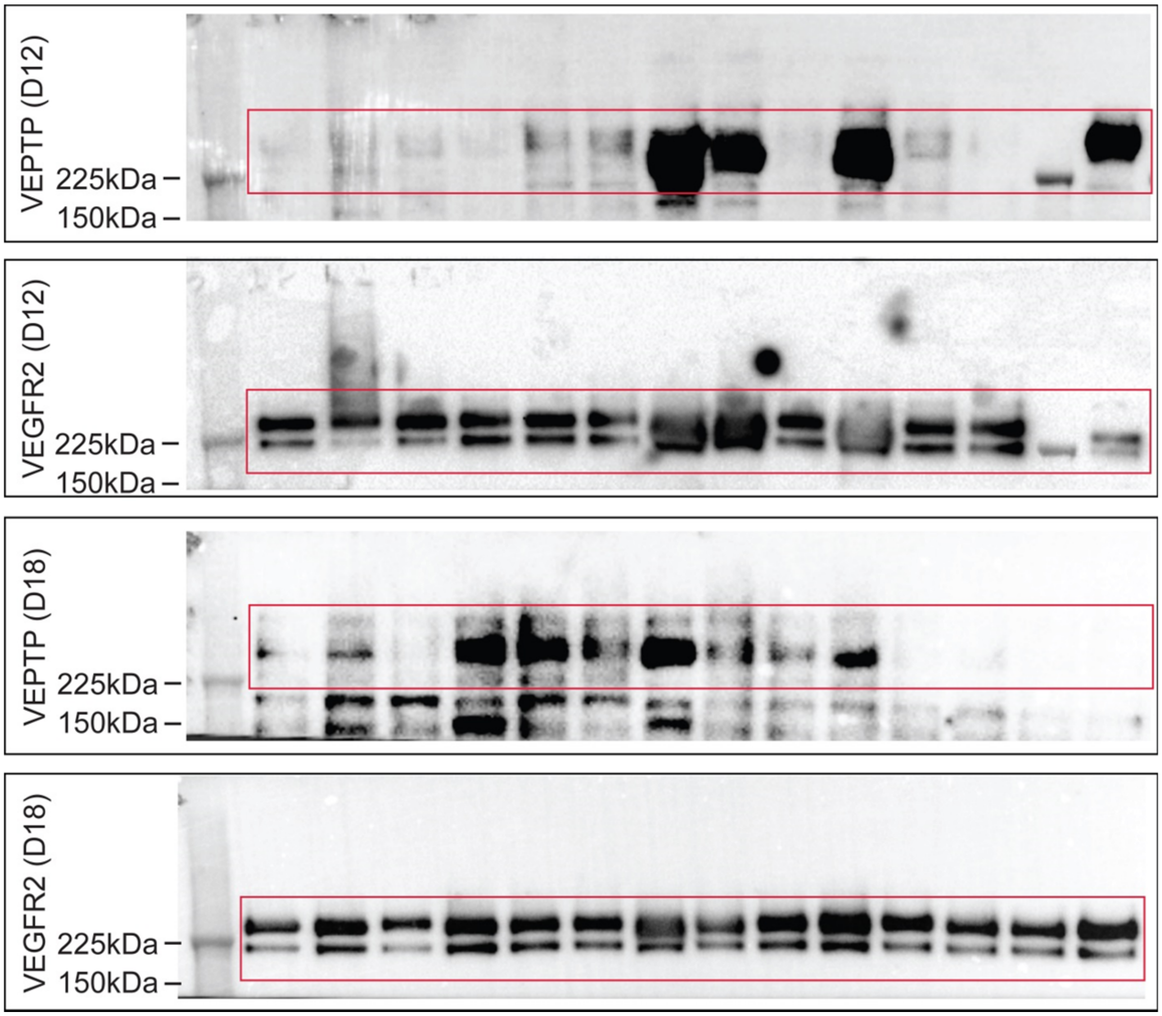

Supplementary Fig. 12. Uncropped immunoblots complementing blots shown in Fig. 6e.

Panels used in Fig. 6e are marked with a red square. B16F10 melanomas from WT or *Vegfr2*$^{Y949F/Y949F}$ mice were harvested at day 12 (D12) or D18 after inoculation, lysed and used for immunoprecipitation of VEGFR2 followed by immunoblotting for VEPTP (D12, uppermost), VEGFR2 (D12, upper middle), VEPTTP (D18, lower middle) and VEGFR2 (D18, lowermost). Molecular weight markers are indicated to the left. See Fig. 6e in Li, Padhan et al. for details.

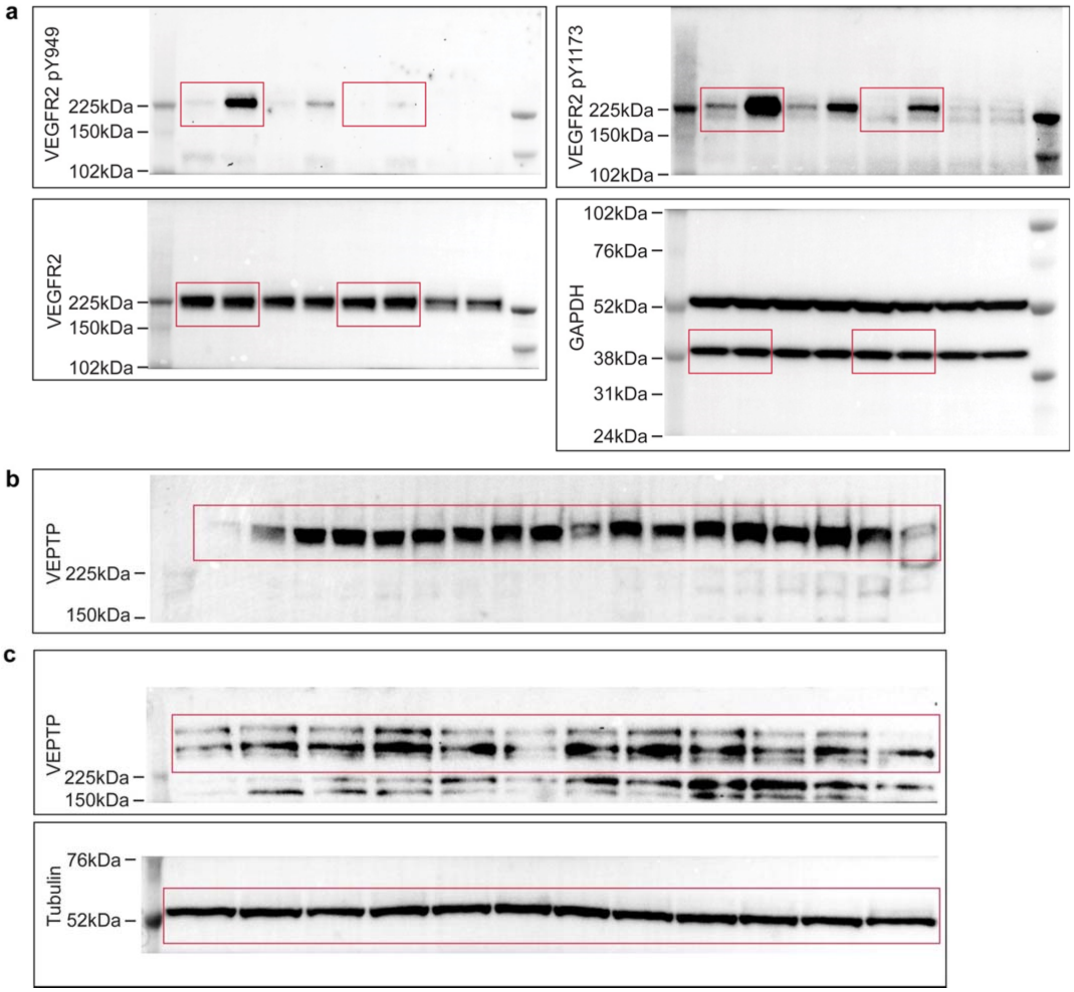

Supplementary Fig. 13. Uncropped immunoblots complementing blots shown in Suppl. Fig. 5.

Cropped areas used for immunblots show in Suppl. Fig. 5a (labeled a), Suppl. Fig. 5b (labeled b) and Suppl. Fig. 5c (labeled c) are marked by red boxes. Antibodies used for immunoblotting are indicated to the far left and molecular markers to the left of each blot. See Suppl. Fig. 5 for details.

Supplementary Table 1. Antibodies used in the study

Antibody name	Manufacturer	Cat No.	Dilution/Conc
**Immunostaining**			
CD31 (Rat)	BD	553370	1:500
CD31 (Armenian Hamster)	Thermo Fischer Scientific	MA3105	1:500
VE-Cadherin	R&D	AF1002	1:200
Podocalyxin	R&D	AF1556	1:200
Type IV collagen	Millipore	AB769	1:50
NG2	Millipore	AB5320	1:200
Desmin	Abcam	ab8592	1:100
ERG1	Abcam	ab92513	1:200
LYVE-1	ReliaTech GmbH	103-PA50	1:50
FITC-conjugated *Lycopersicon esculentum* (tomato) lectin	Vector Laboratories	FL-1171	1:2
Biotinylated *Lycopersicon esculentum* (tomato) lectin	Vector Laboratories	B-1175	1:2
Alexa Fluor® 594-Conjugated *Griffonia simplicifolia*, Isolectin GS-IB_4	Thermo Fisher Scientific	I21413	1:500
Fibrinogen	Nordic Immunological Lab	GAM/Fbg/7S	1:250
CD45	R&D	AF114	1:200
F4/80	AbD Serotec	MCA497G	1:200
MMR	R&D	AF2535	1:500
SV40 Large T Antigen Antibody	Kind gift from Doug Hanahan		1:1000
**Immunoprecipitation**			
VEGFR2	R&D	AF644	1.5 µg/ml
**Westernblot**			
VEGFR2	Cell Signaling	2479	1:2000
VEGFR2 pY949	Cell Signaling	2471	1:1000
VEGFR2 pY1173	Cell Signaling	2478	1:1000
VE-cadherinpY658	In-house production, Dejana lab		1:1000
VE-cadherin	R&D	AF1002	1:500
SrcpY418	Cell Signaling	2101	1:1000
Src	Cell Signaling	2123	1:1000
AktpT308	Cell Signaling	4056	1:1000
Akt	Santa Cruz Biotechnology	sc-8312	1:1000
TSAd	R&D	AF6265	1:1000
PV1	Developmental Studies Hybridoma Bank	None	1:200
VEPTP	In-house production, Vestweber lab	VEPTP 1-8	1:2000
GAPDH	Millipore	MAB374	1:1500
α-Tubulin	Sigma	T9026	1:1000
